# Efficacy of Antioxidant Supplementation to Non-Surgical Periodontal Therapy on Metabolic Control in Type 2 Diabetes Patients: A Network Meta-Analysis

**DOI:** 10.3390/antiox11040621

**Published:** 2022-03-24

**Authors:** Elisa Grillo Araújo, Domitilla Marchiori Sant’Anna Leal de Oliveira, Carolina Castro Martins, Cristine Miron Stefani

**Affiliations:** 1 School of Dentistry, University of Brasilia, Brasilia 70910-900, DF, Brazil; araujo.elisa@aluno.unb.br (E.G.A.); domitilla.oliveira@aluno.unb.br (D.M.S.L.d.O.); 2 School of Dentistry, Federal University of Minas Gerais, Belo Horizonte 31270-901, MG, Brazil; carolcm@ufmg.br

**Keywords:** antioxidants, type 2 diabetes mellitus, non-surgical periodontal therapy

## Abstract

This network meta-analysis (NMA) investigated the effectiveness of antioxidants as adjuncts to non-surgical periodontal therapy (NSPT) in the glycated hemoglobin (HbA1c) control of type 2 diabetes (T2D) patients with periodontitis. PubMed, Cochrane, LILACS, Web of Science, Scopus, Embase, LIVIVO, and grey literature were searched. Risk of bias was assessed with the RoB v2.0 tool. A frequentist NMA assessed HbA1c improvement, through standardized mean difference under a random-effects model. Certainty of evidence was addressed through the GRADE (Grading of Recommendations, Assessment, Development and Evaluations) partially contextualized framework. Ten randomized controlled clinical trials were included, with 234 patients receiving alpha lipoic acid (ALA), cranberry juice, cranberry juice enriched with omega-3, fenugreek, ginger, grape seed, lycopene, melatonin, omega-3, propolis or vitamin C supplementation to NSPT, and 220 patients receiving NSPT alone or with placebo. Nine studies were meta-analyzed. HbA1c improved when NSPT was combined with propolis, ALA and melatonin supplementation (moderate-to-low certainty), compared to NSPT alone or with placebo. Risk of bias issues were found in eight studies. In conclusion, the use of propolis supplementation to NSPT probably results in HbA1c improvement in T2D patients with periodontitis (large effect with moderate certainty), while ALA and melatonin supplementation may contribute to reduce the HbA1c in T2D patients with periodontitis (large effects with low certainty).

## 1. Introduction

Periodontitis and diabetes mellitus are common chronic diseases worldwide. Periodontitis is a multifactorial inflammatory disease associated with dysbiotic plaque biofilms and is characterized by the progressive destruction of the tooth-supporting apparatus [1], while diabetes mellitus is a group of metabolic diseases characterized by hyperglycemia resulting from defects in insulin secretion, insulin action, or both [2].

The three main types of diabetes are type 1 diabetes mellitus, type 2 diabetes mellitus (T2D), and gestational diabetes mellitus, among which T2D accounts for approximately 90% of all diabetes cases [3,4,5]. Adoption of appropriate diet, exercise behaviors and adherence to medication regimens will result in tighter glycemic control that, along with controlled blood pressure and blood lipids, will greatly reduce the burden of diabetes complications [5].

Chronic complications of diabetes are broadly divided into microvascular and macrovascular, with the former having much higher prevalence than the latter. Microvascular complications include neuropathy, nephropathy, and retinopathy, while macrovascular complications consist of cardiovascular disease, stroke, and peripheral artery disease. Finally, there are other complications of diabetes that cannot be included in the two categories, such as periodontitis, reduced resistance to infections, and birth complications among women with gestational diabetes [5,6].

The bidirectional pathogenic association between periodontitis and diabetes has been extensively documented [7,8,9,10]. While diabetes mellitus is associated with increased occurrence and progression of periodontitis, this one is associated with poorer glycemic control [9] and is considered the “sixth complication of diabetes” [11]. The American Diabetes Association has officially recognized this association and recommends screening for periodontal disease as part of a physician’s examination [12].

The oxidative stress has been suggested as an underlying mechanism contributing to periodontitis in patients with T2D, being an important pathogenic factor in this composite disease [13,14,15].

Oxidative stress results from excessive reactive oxygen species (ROS) generation and consists in an imbalance of oxidative to reducing species, being also better defined as a perturbation of redox signaling that results in alterations and function modulations of key biomolecules [16].

The imbalance between ROS and the antioxidant system may contribute to functional and structural remodeling that favors the occurrence of periodontitis [17]. On the other hand, the increased generation of ROS is a potent culprit in diabetes mellitus by inducing β-cell dysfunctions and insulin resistance. Furthermore, oxidative stress is closely related with diabetic complications that are responsible for both the death and long-term disability of patients with diabetes [4].

Studies evaluating proteins, DNA or lipid oxidation end products, antioxidant markers or enzymatic antioxidant mechanisms and using different methods of analysis confirm a consistent link between type 2 diabetes and periodontal disease in terms of the overproduction of ROS and their downstream effects [13,15,18,19,20,21,22].

The inflammatory mediators linked to both T2D and periodontitis, such as interleukin-1-beta, interleukin-6 and tumor necrosis factor-alpha, contribute to the generation of ROS. Moreover, the hyperglycemia induces further generation of ROS. In the presence of a defective antioxidant defense system, either due to endogenous alteration or exogenous inadequacy, the balance tilts in favor of free radicals and oxidative stress develops [23].

Oxidative stress is considered one of the major pathogenetic factors of many oral diseases, such as xerostomia, periodontitis, burning mouth syndrome and oral cancer. Excess of ROS disturbs the natural redox balance of the oral cavity, leading to protein, lipid, and DNA damage [24]. The severity of tissue destruction is higher when periodontal disease is associated with T2D, confirming that oxidative stress is a common factor involved in this tissue loss [13].

Periodontal therapy is based on a clear concept of pathogenesis, involving bacteria as the root cause of periodontal disease. Deposits on the tooth root surfaces may range from soft plaque to hard tenacious calculus. The non-surgical periodontal therapy (NSPT) involves the mechanical removal of these deposits from the root surfaces to establish and maintain periodontal health [25].

Several studies have described the effect of NSPT on glycemic control in patients with T2D and periodontitis [26,27,28,29,30]. NSPT contributes to reduce general inflammatory load as well as a reduction in glycated hemoglobin (HbA1c) levels and, therefore, should be considered as a component of the medical management (i.e., along with other therapeutic and preventive measures) to T2D patients [29].

Some antioxidant sources are currently used in various auxiliary treatments for many diseases [31]. The concept of antioxidant refers to any compound that, when present at a lower concentration compared to that of an oxidizable substrate, can either delay or prevent the oxidation of the substrate [32]. Antioxidant functions imply lowering oxidative stress, DNA mutations, malignant transformations, as well as other parameters of cell damage. Epidemiological studies proved antioxidants’ ability to contain the effects of reactive oxygen species activity and diminish the incidence of diseases [16].

Antioxidant substances perform a preventive role in protecting against the generation of free radicals and therefore natural based antioxidants are one of the more valuable therapeutic agents to reduce the illnesses triggered by oxidative stress [33].

Bearing in mind that the total antioxidant status plays a role in metabolic control and tissue destruction, supplementation with antioxidants as an adjuvant to NSPT in T2D patients may be helpful [24,34].

Different substances were tested for this purpose, but only one meta-analysis, carried out by Mizutani et al., 2021 [35], compared their effects on the clinical periodontal parameters, while no current systematic review evaluated the improvement in metabolic control after antioxidant supplementation as an adjunct to NSPT.

Thus, this systematic review aims to assess whether the adjunctive use of antioxidant supplementation to NSPT results in increased metabolic control in patients with T2D and periodontitis.

## 2. Materials and Methods

### 2.1. Protocol and Registration

This systematic review was carried out according to the PRISMA Extension Statement for Reporting of Systematic Reviews Incorporating Network Meta-analyses of Health Care Interventions [36]. It was registered under the numbers CRD42020207860 at PROSPERO website (international prospective register of systematic reviews, available at https://www.crd.york.ac.uk/prospero/ accessed on 5 September 2020) and the identifier doi:10.17605/OSF.IO/VS8KH in the Open Science Framework website (OSF, available at https://osf.io/ accessed on 7 September 2020). The acronym PICOS was applied to determine the focused question: in patients with T2D and periodontitis (P), does the use of antioxidants as adjuvant to non-surgical periodontal therapy (I) result in increased metabolic control (O) when compared to conventional non-surgical periodontal therapy alone (C)?

### 2.2. Search Strategy and Eligibility Criteria

The first search was carried out on 30 June 2020, and updated on 6 January 2022, using antioxidants, diabetes mellitus type 2 and non-surgical periodontal therapy as descriptors. The MeSH Terms used were: (“Diabetes Mellitus” OR “Glycated Hemoglobin A”) AND (“Antioxidants”) AND (“Chronic Periodontitis” OR “Periodontitis” OR “periodontal debridement”). Word variations and synonyms were also used. The complete search strategy for each database is available at Supplementary File S1. No restrictions on language or publication period were established. Inclusion criteria were:

- (P) adult patients with diagnosed T2D (controlled or not) under treatment (including diet, exercises, pharmacological therapy or any combination of those) and untreated periodontitis (according to the case definition of the new Periodontal Diseases Classification [37], patients with interdental clinical attachment level (CAL) detectable at ≥2 non-adjacent teeth, or buccal or oral CAL ≥ 3 mm with pocketing > 3 mm detectable at ≥2 teeth);
- (I) NSPT with any type of adjunctive antioxidant supplement ingestion;
- (C) NSPT alone or associated to placebo ingestion;
- (O) Metabolic control evaluated through HbA1c level change from baseline;
- (S) Randomized controlled clinical trials (RCTs). Only RCTs were included once this is the most appropriate type of study to answer interventional questions and constitute the best scientific evidence to support the therapeutic practice.

Exclusion criteria comprised books, chapters, editorials, review articles, opinion articles, technical articles, guidelines, observational studies, clinical cases and case-series, non-randomized clinical trials, animal studies and in vitro studies. Studies with samples including type I diabetes patients or children and adolescents, studies in which the control group remained untreated, and studies not evaluating glycated hemoglobin as an outcome were also excluded.

PubMed (MEDLINE), Cochrane (CENTRAL), LILACS (BVS), Web of Science, Scopus, Embase and LIVIVO databases were searched. Additionally, grey literature was searched through ProQuest (Dissertation and Theses), OpenGrey and Google Scholar. Clinical Trials registry (available at https://clinicaltrials.gov/ accessed on 6 January 2022) and hand search of reference list from included studies were analyzed for additional references.

All results were imported into the reference manager Mendeley Desktop software (v1.19.8, Elsevier, Amsterdam, The Netherlands), where duplicate studies were identified and removed. Titles and abstracts were evaluated by two independent reviewers (DMSLO and EGA) according to eligibility criteria in Rayyan QCRI application [38]. Then, studies’ full texts were also analyzed independently to confirm eligibility. Disagreements were solved by a third evaluator (CMS).

### 2.3. Data Extraction and Risk of Bias

Two independent reviewers (DMSLO and EGA) extracted data. Discrepancies were solved by a third reviewer (CMS). Data extracted comprised authors, date of publication, country, participants (sample size and mean age), diabetes and periodontitis case definitions, type of antioxidant supplement and adopted regimen for treated group, treatment delivered to the control group, follow-up, results for glycated hemoglobin, assessed before and after treatment for both groups (treated and control) and statistical analysis.

The risk of bias was performed through the Cochrane’s risk of bias tool for RCTs (RoB v2.0) [39] considering the “per protocol” approach for HbA1c level outcome. The risk of bias was assessed independently by two reviewers (DMSLO and EGA) on five domains (randomization process, deviations from intended interventions, missing outcome data, measurement of the outcome and selection of the reported result) as “low risk”, “some concerns” or “high risk” and disagreement were once more checked by a third evaluator (CMS).

### 2.4. Data Synthesis and Meta-Analysis

HbA1c level change from baseline to 8 or 12 weeks after treatment mean scores (and standard deviations) were calculated to every included study. A frequentist network meta-analysis (NMA) was performed through MetaInsight software v1.1 [40] for continuous variables (available on https://crsu.shinyapps.io/metainsightc/ accessed on 10 October 2021) to compare different antioxidants as adjuncts to NSPT. Random effects method model and inverse variance statistics were used to calculate standardized mean difference with 95% confidence interval.

### 2.5. Certainty of Evidence Assessment

The certainty of evidence was assessed by two reviewers (CMS and CCM) following the GRADE approach with Partially Contextualized Framework for Network Meta-analysis for interpretation of results [41,42] and is available on Supplementary Table S1. For direct comparisons, risk of bias, inconsistency, indirectness, and publication bias were evaluated. For indirect comparisons, first-order loop comparison with the lowest certainty was considered and intransitivity evaluated. Incoherence and imprecision were assessed for the NMA effect estimate. Then, the Partially Contextualized Framework considered the magnitude of the effect and the certainty of the evidence for interpretation of the results [43]. The magnitude of the effect was interpreted according to Cohen’s classification [44]: from −0.2 to 0.2 (trivial or no effect), −0.5 to −0.2 or 0.2 to 0.5 (small effect), −0.8 to −0.5 or 0.5 to 0.8 (moderate effect), or <−0.8 or >0.8 (large effect). The large effect was the threshold to consider a treatment effective. Detailed information on judgment criteria is available on Supplementary File S5.

## 3. Results

### 3.1. Characteristics of the Included Studies

**Figure 1:**
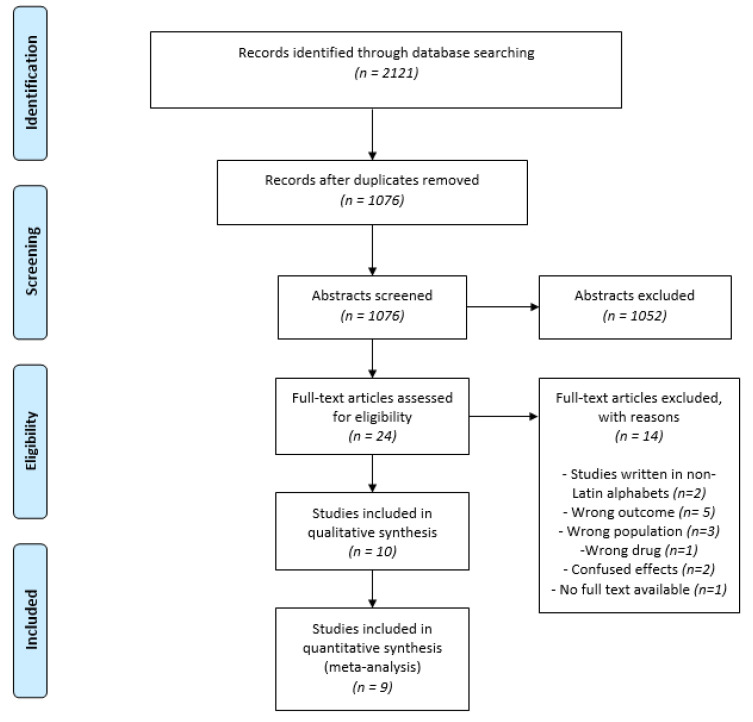
Flow diagram of inclusion process according to the PRISMA statement.

A total of 2121 references were retrieved from database search, resulting in 1076 after removing duplicates. All 1076 articles were analyzed by titles and abstracts, according to the eligibility criteria, and then 1052 were excluded. Twenty-four full texts were read and fourteen were excluded, leaving 10 included studies (Figure 1). A list of excluded articles and reasons for exclusion are available on Supplementary File S2.

Authors, date of publication, country, participants (number and average age), type of antioxidant supplement and dose, results regarding glycated hemoglobin before and after treatment for both groups (treated and control), follow-up and statistical analysis were described in Table 1.

The articles were published between 2015 and 2021 and most of them were conducted in Asian countries (India [45,46,47,48,49], Iran [50,51], Thailand [52], and Egypt [53]), except one, which was performed in Romania [54]. In all included studies, a periodontal clinical examination was conducted to confirm the diagnosis of periodontitis, while the diagnosis of T2D was confirmed by laboratorial tests for HbA1c level. Regarding anti-glycemic therapy, only the studies that evaluated omega-3, ALA and fenugreek specified the concomitant use of metformin 500 mg/day by oral route [46,48,49].

The intervention in the test groups (TGs) was NSPT plus antioxidants and in the control groups (CGs) it was NSPT alone [47,48,49,51] or associated to placebo [45,46,50,52,53,54].

Considering all included studies, 20 patients were treated with alpha lipoic acid (ALA) [49], 40 with fenugreek [48], 20 with lycopene [47], 24 with omega-3 fatty acid [46,51], 9 with cranberry juice [51], 10 with cranberry juice enriched with omega-3 [51], 15 with vitamin C [52], 24 with propolis [53], 21 with ginger [50], 24 with grape seed extracts [45] and 27 with melatonin [54]. In control groups, 92 patients underwent NSPT alone [47,48,49,51], while 128 received placebo together with NSPT [45,46,50,52,53,54].

Excepting one study [51], all the others evaluated only one antioxidant each. Only the Zare-Javid et al. [51] study included four arms, testing two different antioxidants (cranberry and omega-3), alone or in combination, compared to NSPT.

Rampally et al. [46], in addition to the effects of the antioxidant (omega-3 fatty acids), also evaluated low-dose aspirin, but only the data regarding the antioxidant were considered.

Besides HbA1c, studies evaluated other biochemical measurements: pentraxin (PTX3) [46], fast blood glucose (FBG) [48,50,51,52,53], lipid profile (LDL-C, HDL-C, TC, TG) [50,51], resistin [49], malondialdehyde (MDA) [47,50], C-reative protein (CRP) [47], N-carboxymethyl lysine (CML) [53], plasma vitamin C [52], interleukin-6 (IL-6) [48], total antioxidant capacity (TAOC) [45] and myeloperoxidase (MPO) [45]. One of them [51] also assessed anthropometric and nutritional aspects.

### 3.2. Risk of Bias

**Figure 2:**
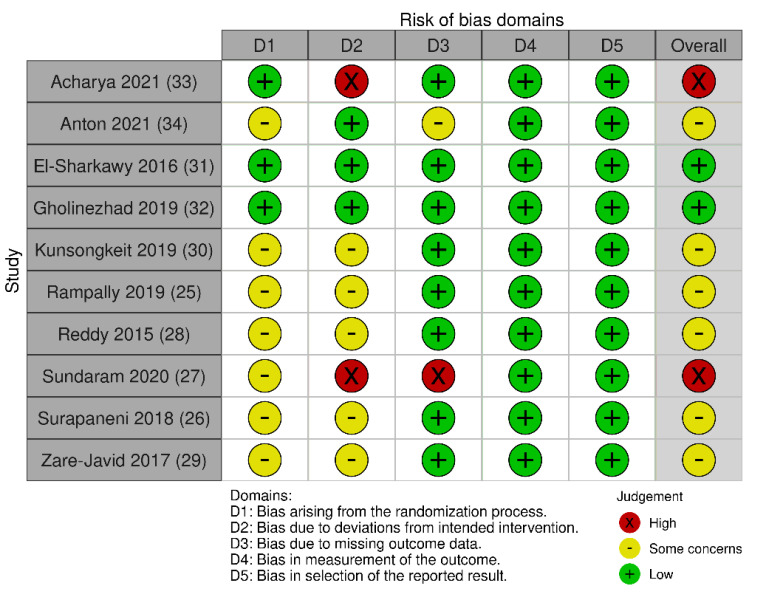
Traffic light plot of risk of bias assessment of included studies for each RoB v2.0. tool domain, and overall risk of bias.

**Figure 3:**
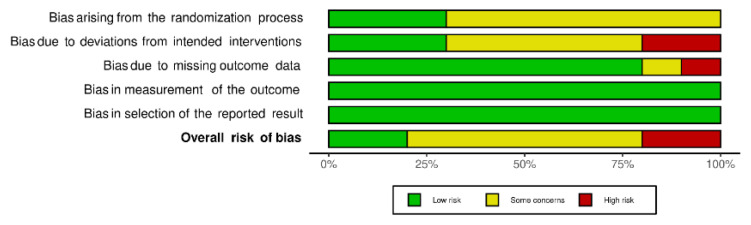
Weighted bars plot of risk of bias evaluation of included studies (Cochrane’s RoB v2.0 tool).

Two studies [50,53] were considered at “low” risk of bias, six studies [46,47,49,51,52,54] presented “some concerns” and two studies [45,48] were identified as “high” risk of bias (Figure 2 and Figure 3).

In the studies with “some concerns” [46,47,49,51,52,54], the risk of bias was mainly due to the lack of information about the randomization process and concealment of the allocation sequence, or the lack of operator blinding. One study was classified as “high” risk of bias [48], and in addition to the questions mentioned previously, it did not report missing outcome data. The other study [45] did not mention adherence to the intended intervention verification strategy.

### 3.3. Network Meta-Analysis Results

**Figure 4:**
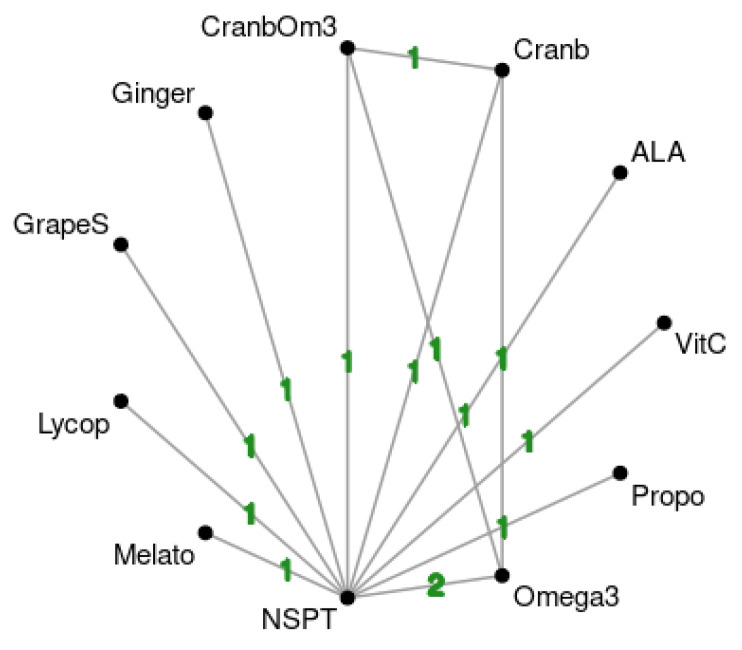
Network plot for included studies. Numbers in lines show number of included studies in direct comparisons (ALA: alpha lipoic acid; Cranb: cranberry; CranbOm3: cranberry plus omega-3; GrapeS: grape seed; Lycop: lycopene; Melato: melatonin; NSPT: nonsurgical periodontal therapy: Propo: propolis; VitC: vitamin C).

**Figure 5:**
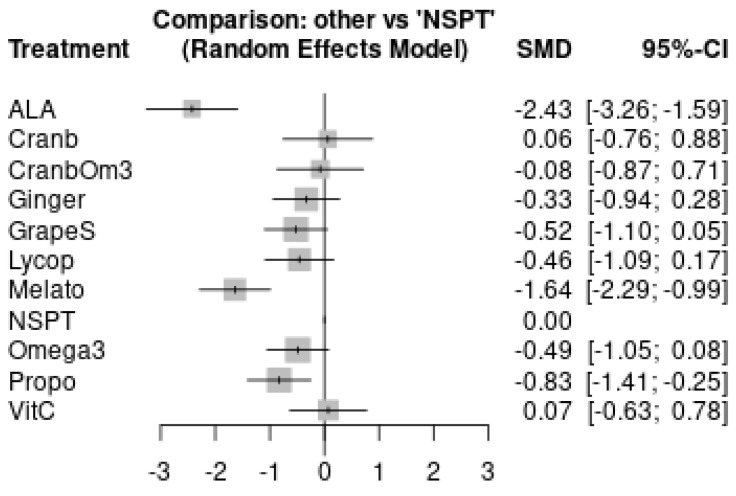
Forest plot showing the performance of different antioxidants used as adjuncts to NSPT, compared to NSPT alone.

One study was excluded from meta-analysis due to insufficient follow-up [48] (4 weeks). Network plot is shown in Figure 4. It is possible to notice that comparison of included studies resulted in a poorly connected network. The league table for all comparisons’ effect estimates is available in Supplementary File S3. Table 2 presents the interpretation of the results using the GRADE partially contextualized framework. Propolis was the most effective treatment when compared to NSPT with moderate certainty (−0.83; 95%CI: −1.41, −0,25). ALA (−2.43; 95%CI: −3.26, −1.59) and melatonin (−1.64; 95%CI: −2.29, −0.99) had low certainty of evidence of effectiveness when compared to NSPT. The other treatments had very low certainty of evidence (grade seeds, omega-3, lycopene, ginger, cranberry + omega-3, cranberry, vitamin C). The forest plot comparing all antioxidant groups and NSPT alone is shown in Figure 5. Inconsistency test results are shown in Supplementary File S4.

### 3.4. Certainty of Evidence

Certainty of evidence, evaluated through GRADE partially contextualized framework for NMA [41,42], varied from moderate to very low (Supplementary File S3 and Table 2). Downgrading was mainly due to risk of bias, indirectness, intransitivity, incoherence, and imprecision. Detailed results for each comparison judgment are available on Supplementary File S5.

## 4. Discussion

The results show that propolis supplementation to NSPT was the most effective treatment resulting in HbA1c improvement, when compared to NSPT alone. ALA and melatonin, like propolis, had a large effect size for the intervention, yet only propolis had moderate certainty of evidence. Risk of bias and imprecision were the main factors contributing to decrease the level of certainty of ALA and melatonin. All other antioxidants had a similar effect when compared to NSTP (Table 2).

Propolis, also known as bee glue, is a non-toxic resin material produced by bees that presents several interesting properties, including antioxidant, antimicrobial, anti-inflammatory, antitumor, antiviral, antifungal, antihypertensive, antiplatelet, and immune-stimulating effects [55,56,57]. Because of its antimicrobial activity, propolis is also called a natural antibiotic [58]. Polyphenols, substances found in propolis, have been suggested as effective compounds that might prevent and manage T2D, increasing glucose metabolism, decreasing insulin resistance and HbA1c level, and improving vascular function [59]. Furthermore, these compounds improve oxidative stress indices and can help to reduce the complications of T2D [60]. Two recent meta-analyses showed a significant reduction in HbA1c and fasting plasma glucose (FPG) after propolis supplementation [55,57]. This reduction may be attributed to propolis’ ability to promote glucose uptake, increase insulin production and/or enhance cellular sensitivity to this hormone [55]. However, other studies have contradictory results, showing no improvement in glycemic status [61,62]. Discrepancies among evidence might be related to differences in the duration of supplementation, dosage, population characteristics and the sample size of trials [57]. Another issue is the source of propolis, since its compounds are highly affected by geographic area, environmental factors, and beekeeper actions [63].

There is also evidence that propolis can be beneficial in periodontitis’ treatment, improving the results of NSPT due its anti-inflammatory, antibacterial and antioxidant properties [56,58]. The use of this substance could reduce the prevalence of periodontal pathogens (*Porphyromonas gingivalis*, *Prevotella intermedia* and *Fusobacterium nucleatum*), in addition to potentially improving periodontal parameters when used as an adjunct to NSPT [58]. According to a recent systematic review [64], the properties of propolis also improve bone remodeling by increasing osteoblastogenesis and decreasing osteoclastogenesis. This skeletal protective effect may inhibit bone loss due to periodontitis [64]. In the present NMA, the comparison of propolis supplementation to NSPT showed a large effect when compared to NSPT alone in reducing HbA1c. Despite only a single study testing this antioxidant being found in the literature [53], it was considered to be at “low” risk of bias, resulting in a moderate evidence certainty, confirming previous studies, and therefore suggesting that propolis supplementation could be beneficial for T2D patients with periodontitis.

ALA supplementation to NSPT presented the largest effect size when compared to NSPT alone and most tested antioxidants; however, this was with low-to-very-low certainty. Previous studies evaluating the effect of ALA on both conditions, periodontitis and diabetes, separately, showed promising results [65,66]. Non-diabetic patients using ALA as an adjunct to periodontal treatment showed clinical periodontal parameter improvement through its antioxidant and alveolar bone protective effects, beyond the ability to inhibit inflammation mediators and bind metals, reconciling bone tissue metabolism [65]. In diabetes treatment, ALA has been shown to prevent beta cell destruction and enhance glucose uptake, while its antioxidant effects may be beneficial in reducing the development of diabetic complications, mainly diabetic neuropathy [66]. Those previous positive results related to the use of ALA in periodontal therapy and in the treatment of diabetes suggested that this substance could improve T2D patients with periodontitis conditions. In fact, the results found were quite promising; however, with low certainty. Only one study evaluated the use of ALA [49] in T2D patients with periodontitis, and it presented some concerns about the randomization process and blinding description of participants and operators. Furthermore, the small number of patients in the comparison resulted in rating down due to imprecision.

Melatonin was another antioxidant that presented a large effect size with low certainty of evidence in the NMA, when compared to NSPT alone. Despite previous studies demonstrating its effects in diabetes and periodontitis’ treatment [67,68,69], the search returned only one RCT evaluating the melatonin supplementation as an adjunct to NSPT in T2D patients with periodontitis [54], with some concerns regarding the randomization process and small sample, resulting in a suboptimal information size for the comparison. In a recent systematic review with meta-analysis [35], a significant improvement in periodontal parameters was reported in T2D patients who received melatonin associated with NSPT, when compared to NSPT alone or plus placebo. However, effects of melatonin supplementation on glycemic status were not addressed. It is noteworthy that the review included RCTs with important methodological inconsistencies; in addition to this, substances with different use protocols (ingestion or local gel application) were compared. Moreover, the certainty of evidence was not assessed. Therefore, those results must be interpreted with caution.

### 4.1. Limitations and Strengths

This review has limitations, namely, performing a subgroup analysis was an impossibility due to the small number of studies. Moreover, the small number of studies with longer follow-up times made it impossible to perform a meta-regression or a subgroup analysis, since only three studies reached 24 weeks follow-up [45,47,53]. Additionally, the small number of RCT testing antioxidants as an adjunct to NSPT resulted in a poorly connected network; consequently, for some comparisons, only direct comparisons were possible, with no indirect possibilities.

This study’s strengths comprise the NMA that allows one to establish a comparison between several treatments that were not directly compared in a study and evaluate the effectiveness of each intervention, estimating which one is the best for each outcome [70]. Another strength of this study is its highly rigorous methodological approach—that is, using the GRADE partially contextualized framework to assess the certainty of the evidence and interpret the results. Moreover, this framework together with decision thresholds to interpret results of NMA is more conservative and can avoid misinterpretations and misleading results [43].

### 4.2. Implications for Future Research and Clinical Practice

Given the deficiencies of the current evidence identified in this review, future studies might include: (1) inclusion criteria carefully elaborated, with the diabetes case definition clearly described; (2) measures to reduce the risk of bias and methods accurately described in the study report, with special attention to randomization, allocation concealment, and blinding of patients and evaluators; (3) larger sample size and repeated measurements of HbA1c levels to reduce imprecision; (4) longer follow-up periods.

Regarding the clinical practice, the indication of antioxidant supplementation as an NSPT adjunct is still premature, since more robust evidence is necessary to endorse it. Further larger and longer high-quality intervention trials are needed to confirm the efficacy of the various antioxidant substances available, as well as to determine the best antioxidant consumption protocol.

## 5. Conclusions

The use of propolis supplementation as an adjunct to the NSPT probably results in HbA1c control improvement in T2D patients with periodontitis (large effect with moderate certainty), while ALA and melatonin supplementation may contribute to reduce the HbA1c in T2D patients with periodontitis (large effects with low certainty).

## Figures and Tables

**Table 1:** Summary of descriptive characteristics of included articles (*n* = 10).

Author, Year Country	Age in Years Mean ± SD and/or Range	Case Definitions	Groups (*N*)	Treatments TG CG	Baseline HbA1c % Mean ± SD	Follow-Up (in Months)	Final HbA1c % Mean ± SD (*p* Value)	Main Conclusions
Acharya et al., 2021, India	NA	Periodontitis: CP with PPD ≥ 5 mm Diabetes: HbA1c in the range 6%–8% and FBS in the range 135–205 mg/dL, for 5 to 10 years	TG (*n* = 24) CG (*n* = 24)	TG: 200 mg of Grape Seed extract for 12 weeks + NSPT CG: placebo for 12 weeks + NSPT	TG 7.33 ± 0.73 CG 7.3 ± 0.71	12 weeks 24 weeks	TG 6.38 ± 0.51 (*p* < 0.01) CG 6.81 ± 0.55 (*p* < 0.01) CBG: NS TG 6.68 ± 0.59 (*p* < 0.01) CG 6.76 ± 0.54 (*p* < 0.01) CBG: NS	This study shows a promising result in using grape seed formulation as an adjunct to scaling and root planing to reduce the oxidative stress, decreasing the inflammation and achieving the glycaemic control in diabetic patients with CP.
Anton et al., 2021, Romania	TG 53.24 ± 3.4 CG 52.21 ± 3.1	Periodontitis: CAL ≥ 5 mm Diabetes: FBS > 126 mg/dL and HbA1c > 6.5%	TG (*n*= 27) CG (*n*= 27)	TG: two tablets containing 3 mg of melatonin daily for 8 weeks + NSPT CG: placebo for 8 weeks + NSPT	TG 7.62 ± 0.71 CG 7.61 ± 0.62	8 weeks	TG 6.28± 0.31 *p* < 0.001 CG 7.58 ± 0.57 (NS) CBG: *p* < 0.001	Combined NSPT and systemic treatment with melatonin provided additional improvements to severe periodontal condition (improve PPD and CAL) and the glycemic control of patients with type 2 diabetes.
El-Sharkawy et al., 2016, Egypt	TG 48.9 ± 8.3 Age range: 38–63 CG 51.2 ± 6.5 Age range: 40–61	Periodontitis: PPD and CAL ≥ 5 mm with BOP in at least one site in each sextant Diabetes: History T2D > 5 years	TG (*n* = 24) CG (*n* = 26)	TG: 400 mg propolis capsule orally daily for 24 weeks + NSPT CG: placebo for 24 weeks + NSPT	TG 8.73 ± 0.55 CG 8.59 ± 0.91	12 weeks 24 weeks	TG: 8.71 ± 0.56 (*p* < 0.01) CG: 8.58 ± 0.82 (NS) CBG: NA TG 7.75 ± 0.48 (*p* < 0.01) CG 8.5 ± 0.73 (NS) CBG: NA	A 6-month regimen of 400 mg daily propolis + SRP significantly reduces HbA1c levels and improves periodontal therapy outcomes (PPD and CAL gain).
Gholinezhad et al., 2019, Iran	TG 52.81 ± 6.44 CG 51.62 ± 5.95	Periodontitis: PPD ≥ 4 mm and CAL = 1–4 mm Diabetes: FBS ≥ 126 mg/dL and HbA1c ≥ 6.5% > 5 years	TG (*n* = 21) CG (*n* = 21)	TG: two tablets with 1 g ginger supplement twice daily for 8 weeks + NSPT CG: placebo for 8 weeks + NSPT	TG 8.60 ± 1.37 CG 8.35 ± 1.01	8 weeks	TG 7.84 ± 1.48 (*p* = 0.008) CG 8.18 ± 1.02 (NS) CBG: NS	Ginger + NSPT may be effective in control of the glycemic, lipid, antioxidant, and periodontal status (PPD, CAL, PI and BOP levels) in T2DM patients with CP.
Kunsongkeit et al., 2019, Thailand	TG 59.87 ± 11.3 CG 57.94 ± 14.0	Periodontitis: CAL ≥ 3 mm and PD ≥ 5 mm at least in one tooth Diabetes: FBS > 150 mg/dL and HbA1c > 7%	TG (*n* = 15) CG (*n* = 16)	TG: 500 mg/day vitamin C for 8 weeks + NSPT CG: placebo for 8 weeks + NSPT	TG 7.53 ± 0.79 CG 8.39 ± 1.50	8 weeks	TG 7.27 ± 0.88 (NS) CG 7.98 ± 1.85 (NS) CBG: NS	Supplementation of 500 mg/day vitamin C did not give an additional benefit, HbA1c were not significantly different compared with baseline in the test group. All periodontal parameters were significantly improved in both groups.
Rampally et al., 2019, India	Age range: 30–65	Periodontitis: at least four teeth with one or more sites with PD ≥ 5 mm and CAL ≥ 4 mm Diabetes: HbA1c ≥ 6.5%	TG1 (*n* = 14) TG2 (*n* = 14) CG (*n* = 14)	TG1 75 mg of aspirin orally once a day for 12 weeks NSPT TG2 500 mg of O3FAs orally twice a day for 12 weeks + NSPT CG placebo for 12 weeks + NSPT	TG1 8.97 ± 1.46 TG2 8.079 ± 1.15 CG 7.54 ± 0.82	12 weeks	TG1 6.98 ± 0.88 (*p* < 0.001) TG2 7.136 ± 1.21 (*p* < 0.001) CG 7.25 ± 0.81 (*p* < 0.001) CBG: NS	All groups showed statistically significant results after 3 months for HbA1c and periodontal clinical parameters (GI, PPD and CAL). However, the difference between the groups was not significant for those parameters.
Reddy et al., 2015, India	Age range: 35–50	Periodontitis: at least four teeth with one or more sites with PPD ≥ 5 mm, CAL ≥ 4 mm and BOP Diabetes: FPG >126 mg/dL	TG (*n* = 20) CG (*n* = 20)	TG: 8 mg Lycopene soft gels daily for 8 weeks + NSPT CG: NSPT	TG 7.58 ± 0.88 CG 7.80 ± 0.98	8 weeks 24 weeks	TG 6.10 ± 0.56 (*p* < 0.001) CG 6.84 ± 0.65 (*p* < 0.001) CBG: *p* < 0.001 TG 6.82 ± 0.61 (NS) CG 7.12 ± 0.41 (NS) CBG: NS	Lycopene along NSPT was effective in restoring altered glycemic control and in reducing the PPD in diabetic patients.
Sundaram et.al. 2020, India	NA	Periodontitis: at least 30% of the sites with CAL ≥ 4 mm, PD ≥ 5 mm and BOP Diabetes: HbA1c > 8% and history T2D > 5 years	TG (*n* = 40) CG (*n* = 40)	TG: 12,5 mg fenugreek powder twice daily for 4 weeks + NSPT CG: NSPT	CG 8.5 ± 0.9 TG 8.90 ± 1.1	4 weeks	TG 6.7 ± 0.5 (*p* < 0.001) CG 7.3 ± 0.6 (NS) CBG: NS	Fenugreek + NSPT might have added additional benefit in reducing the glycemic status There was also a significant reduction in the PI.
Surapaneni et al., 2018, India	35–60 (mean age 50.3)	Periodontitis: at least 4 teeth with PPD ≥ 5 mm, CAL ≥ 4 mm and BOP Diabetes: HbA1c ≥ 6.5% up to 10%, recently diagnosed (<1 month)	TG (*n* = 20) CG (*n* = 20)	TG: Alpha Lipoic Acid 600 mg thrice a day for 12 weeks + NSPT CG: NSPT	TG 9.9 ± 0.3 CG 8.6 ± 1.1	12 weeks	TG 6.3 ± 0.3 (*p* < 0.001) CG 7.4 ± 0.7 (*p* < 0.001) CBG: *p* < 0.001	Alpha Lipoic Acid + NSPT proved to be efficacious in improving the clinical parameters (GI, PPD and CAL), and glycemic control in patients with CP and T2DM.
Zare Javid et al., 2017, Iran	TG1: 57,75 ± 8,58 TG2: 57,88 ± 6,03 TG3: 53,14 ± 6,91 CG: 53,60 ± 6,23	Periodontitis: ten selected sites PPD ≥ 4 mm from at least 3 of the quadrants Diabetes: History T2D > 5 years	TG1 (*n* = 10) TG2 (*n* = 9) TG3 (*n* = 10) CG (*n* = 12)	TG1: 1 g O3FA capsule twice daily, for 8 weeks + NSPT TG2: 200 mL cranberry juice twice daily for 8 weeks + NSPT TG3: 200 mL cranberry juice enriched with 1 g O3FA twice daily for 8 weeks + NSPT CG: NSPT	TG1 6.82 ± 1.31 TG2 6.17 ± 0.53 TG3 6.32 ± 0.40 CG 6.64 ± 0.72	8 weeks	TG1 5.95 ± 0.60 (*p* = 0.025) TG2 5.92 ± 0.65 (NS) TG3 5.92 ± 0.19 (*p*= 0.047) CG 6.35 ± 0.76 (NS) CBG: NS	Cranberry juice enriched with O3FA can be beneficial in decreasing HbA1c and improving periodontal status in patients with diabetes and periodontal disease.

Legend: TG: teste group; CG: control group; HbA1c: glycated hemoglobin; NA: not available; FBS: fasting blood sugar; CAL: clinical attachment level; PPD: probing pocket depth; T2D: type 2 diabetes mellitus; NSPT: non-surgical periodontal therapy; BOP: bleeding on probing; GI: gingival index; SBI: sulcus bleeding index; O3FAs: omega-3 fatty acids; PI: plaque index; CBG: comparison between groups; NS: non-significant.

**Table 2:** Classification of 10 interventions for HbA1c control in patients with type 2 diabetes mellitus and periodontitis following the partially contextualized framework for NMA.

Cohen’s Classification ^1^	Intervention ^2^	Intervention versus NSPT SMD ^3^ [95% CI]	Intervention versus NSPT MD ^4^ [95% CI]	Certainty
Large effect	Propolis	−0.83 [−1.41; −0.25]	−0.74 [−1.22; −0.26]	Moderate
Large effect	ALA	−2.43 [−3.26; −1.59]	−2.40 [−3.00; −1.80]	Low
	Melatonin	−1.64 [−2.29; −0.99]	−1.31 [−1.75; −0.87]	Low
Moderate effect	Grape Seeds	−0.52 [−1.10; 0.05]	−0.46 [−0.95; 0.03]	Very Low
Small effect	Omega-3	−0.49 [−1.05; 0.08]	−0.62 [−1.37; 0.14]	Very Low
	Lycopene	−0.46 [−1.09; 0.17]	−0.52 [−1.21; 0.17]	Very Low
	Ginger	−0.33 [−0.94; 0.28]	−0.59 [−1.65; 0.47]	Very Low
Trivial/No effect	Cranberry + Omega-3	−0.10 [−0.94; 0.74]	−0.11 [−0.77; 0.55]	Very Low
	Cranberry	0.04 [−0.83; 0.90]	0.04 [−0.77; 0.85]	Very Low
	Vitamin C	0.07 [−0.63; 0.78]	0.15 [−1.28; 1.58]	Very Low

ALA: alpha lipoic acid; CI: confidence interval; MD, mean difference; NSPT: nonsurgical periodontal therapy; SMD, standardized mean difference. ^1^ From −0.2 to 0.2 (trivial or no effect), −0.5 to −0.2 or 0.2 to 0.5 (small effect), −0.8 to −0.5 or 0.5 to 0.8 (moderate effect), or <−0.8 or >0.8 (large effect). ^2^ Used as an adjunct of NSPT and compared to NSPT alone. ^3^ Results in SD. Negative values mean that the intervention was more effective in reducing HbA1c. Positive values mean that the comparator (NSPT) was more effective. ^4^ Results in % HbA1c. Negative values mean that the intervention was more effective in reducing HbA1c. Positive values mean that the comparator (NSPT) was more effective.

## Supplementary Materials

The following are available online at https://www.mdpi.com/article/10.3390/antiox11040621/s1, Supplementary File S1: Complete search strategy for each database; Supplementary File S2: Excluded articles and reason for exclusion. (*n* = 14); Supplementary File S3: League table showing the effect estimates and 95%CI for each comparison. The lower triangle shows the results of mixed comparisons; the upper triangle shows direct comparisons. Negative results favor treatments showed in the columns, positive results favor treatment in the rows. Bold results indicate that the treatment in the column was more effective compared to the intervention in the line. The colors show the certainty of evidence for each comparison, according to the legend on the lower left (green: high certainty; yellow: moderate certainty; orange: low certainty; light red: very low certainty); Supplementary File S4: Assessment of inconsistency for all studies (SMD); Supplementary File S5: GRADE Approach analysis and explanations; Table S1: Assessment of GRADE for direct and indirect comparisons evidence certainty.
