# Supplementary material for: Efficacy of Antioxidant Supplementation to Non-Surgical Periodontal Therapy on Metabolic Control in Type 2 Diabetes Patients: A Network Meta-Analysis
Source: Antioxidants (Basel). 2022 Mar 24;11(4):621. doi: 10.3390/antiox11040621 (PMC9031448; doi:10.3390/antiox11040621)
Supplement: Supplementary file 1 [file antioxidants-11-00621-s001.zip › antioxidants-1603468-supplementary.pdf]

Supplementary File S1: Complete search strategy for each database

Database	Search<br><br>1st search: 2020-06-30<br><br>2nd search: 2022-01-06	References
**PubMed**	**((((((((((((((((((((((((((((((("Diabetes Mellitus"[All Fields] OR "diabetes mellitus type 2"[All Fields]) OR "Diabetes"[All Fields]) OR "Metabolic Diseases"[All Fields]) OR "Hyperglycemia"[All Fields]) OR "Glucose Metabolism Disorders"[All Fields]) OR "Metabolic Control"[All Fields]) OR "Glycated Hemoglobin A"[All Fields]) OR "HbA1"[All Fields]) OR "Glycosylated Hemoglobin A"[All Fields]) OR "Blood Glucose"[All Fields]) OR "Glycemic Index"[All Fields]) OR "Fasting blood glucose"[All Fields]) OR "Post-prandial blood glucose"[All Fields]) OR "diabetes mellitus type 2"[All Fields]) OR "diabetes type 2"[All Fields]) OR "diabetes mellitus type ii"[All Fields]) OR "type 2 diabetes mellitus"[All Fields]) OR "type II diabetes mellitus"[All Fields]) OR "type 2 diabetes"[All Fields]) OR "type II diabetes"[All Fields]) OR "advanced glycation end products"[All Fields]) OR "HbA1c"[All Fields]) OR "hemoglobin a1c protein human"[All Fields]) OR "glycohemoglobin A"[All Fields]) OR "hemoglobin a glycosylated"[All Fields]) OR ((("Glycated Hemoglobin A"[MeSH Terms] OR "Glycated Hemoglobin A"[All Fields]) OR "hemoglobin a glycosylated"[All Fields]) AND ("analysis"[MeSH Subheading] OR "analysis"[All Fields]))) OR "hemoglobins glycated"[All Fields]) OR "glycated hemoglobin"[All Fields]) OR "blood glucose metabolism"[All Fields]) OR "blood glucose analysis"[All Fields]) AND (("Antioxidants"[All Fields] OR "Antioxidant Effect"[All Fields]) OR "effect antioxidant"[All Fields]) OR "anti oxidant"[All Fields]) OR "anti oxidant"[All Fields]) OR "anti oxidant effects"[All Fields]) OR "anti oxidant effects"[All Fields]) OR "effects anti oxidant"[All Fields]) OR "Antioxidant Effects"[All Fields]) OR "effects antioxidant"[All Fields]) OR "vitamin c"[All Fields]) OR "vitamin c"[All Fields]) OR "ascorbic acid"[All Fields]) OR "ascorbic acid"[All Fields]) OR "acid ascorbic"[All Fields]) OR "l ascorbic acid"[All Fields]) OR "acid l ascorbic"[All Fields]) OR "l ascorbic acid"[All Fields]) OR "vitamin e"[All Fields]) OR "vitamin e"[All Fields]) OR "carotenoid"[All Fields]) OR "beta-carotene"[All Fields]) OR "beta-carotene"[All Fields]) OR "beta-carotene"[All Fields]) OR "flavonoid"[All Fields]) OR "flavanol"[All Fields]) OR "flavanone"[All Fields]) OR "catechin"[All Fields]) OR "isoflavone"[All Fields]) OR "selenium"[All Fields]) OR "anthocyanidin"[All Fields]) OR "anthocyanins"[All Fields]) OR "tocopherols"[All Fields]) OR "alpha-tocopherol"[All Fields]) OR "alphatocopherol"[All Fields]) OR "vitamin a"[All Fields]) OR "vitamin a"[All Fields]) OR "retinol"[All Fields]) OR "pro-vitamin A"[All Fields]) OR "minerals"[All Fields]) OR "antioxidant minerals"[All Fields]) OR "zinc"[All Fields]) OR "copper"[All Fields]) OR "manganese"[All Fields]) OR "Magnesium"[All Fields]) OR "chromium"[All Fields]) OR "coenzyme Q10"[All Fields]) OR "curcumin"[All Fields]) OR "zeaxanthin"[All Fields]) OR "riboflavin"[All Fields]) OR "taurine"[All Fields]) OR "cobalamin"[All Fields]) OR "Resveratrol"[All Fields]) OR "Tea"[All Fields]) OR "Green Tea"[All Fields]) OR "Green Teas"[All Fields]) OR "tea green"[All Fields]) OR "teas green"[All Fields]) OR "Black Tea"[All Fields]) OR "Black Teas"[All Fields]) OR "tea black"[All Fields]) OR "teas black"[All Fields]) OR "Vitamin B Complex"[All Fields]) OR "vitamin b"[All Fields]) OR "vitamin b"[All Fields]) OR "Thiamine"[All Fields]) OR "Pantothenic Acid"[All Fields]) OR "Niacinamide"[All Fields]) OR "Lycopene"[All Fields]) OR "vitamin d"[All Fields]) OR "vitamin d"[All**	1st search 231<br><br>2nd search 274

	**Fields]) OR "Aloe"[All Fields]) OR "Fruit"[All Fields]) OR "Seeds"[All Fields]) OR "cranberry"[All Fields]) OR "cranberry extract"[All Fields]) OR "berries"[All Fields]) OR "lutein"[All Fields]) OR "Phenolic compounds"[All Fields]) OR "quercetin"[All Fields]) OR "Polyphenols"[All Fields]) OR "coumaric acid"[All Fields]) OR "vitamins"[All Fields]) OR "catalase"[All Fields]) OR "glutathione peroxidase"[All Fields]) OR "superoxide dismutase"[All Fields]) OR "acetylcysteine"[All Fields]) OR "N-acetylcysteine"[All Fields]) OR "Melatonin"[All Fields]) OR "omega-3 fatty acid"[All Fields]) OR "tannins"[All Fields])) AND (((((((((((((((((((((((((((((((((("Chronic Periodontitis"[All Fields] OR ((("Chronic Periodontitis"[MeSH Terms] OR ("chronic"[All Fields] AND "Periodontitis"[All Fields])) OR "Chronic Periodontitis"[All Fields]) OR ("chronic"[All Fields] AND "Periodontitides"[All Fields]))) OR ((("Chronic Periodontitis"[MeSH Terms] OR ("chronic"[All Fields] AND "Periodontitis"[All Fields])) OR "Chronic Periodontitis"[All Fields]) OR ("Periodontitides"[All Fields] AND "chronic"[All Fields]))) OR "periodontitis chronic"[All Fields]) OR "Adult Periodontitis"[All Fields]) OR ((("Chronic Periodontitis"[MeSH Terms] OR ("chronic"[All Fields] AND "Periodontitis"[All Fields])) OR "Chronic Periodontitis"[All Fields]) OR ("adult"[All Fields] AND "Periodontitides"[All Fields]))) OR ((("Chronic Periodontitis"[MeSH Terms] OR ("chronic"[All Fields] AND "Periodontitis"[All Fields])) OR "Chronic Periodontitis"[All Fields]) OR ("Periodontitides"[All Fields] AND "adult"[All Fields]))) OR "periodontitis adult"[All Fields]) OR "Periodontal treatment"[All Fields]) OR "Periodontal therapy"[All Fields]) OR "nonsurgical periodontal therapy"[All Fields]) OR "non-surgical periodontal therapy"[All Fields]) OR "Periodontitis"[All Fields]) OR "Periodontitides"[All Fields]) OR "Periodontal Diseases"[All Fields]) OR "disease periodontal"[All Fields]) OR "Periodontal Disease"[All Fields]) OR "Parodontosis"[All Fields]) OR "Parodontoses"[All Fields]) OR "Pyorrhea Alveolaris"[All Fields]) OR "scaling and root planing"[All Fields]) OR "Periodontal Pocket"[All Fields]) OR ((("clinical trials, phase i as topic"[MeSH Terms] OR "phase i as topic clinical trials"[All Fields]) OR "phase 1"[All Fields]) AND ((((((("periodontal"[All Fields] OR "periodontally"[All Fields]) OR "periodontically"[All Fields]) OR "periodontics"[MeSH Terms]) OR "periodontics"[All Fields]) OR "periodontic"[All Fields]) OR "Periodontitis"[MeSH Terms]) OR "Periodontitis"[All Fields]) OR "Periodontitides"[All Fields]) AND (((((("therapeutics"[MeSH Terms] OR "therapeutics"[All Fields]) OR "therapies"[All Fields]) OR "therapy"[MeSH Subheading]) OR "therapy"[All Fields]) OR "therapy s"[All Fields]) OR "therapys"[All Fields]))) OR "initial periodontal therapy"[All Fields]) OR "Periodontal Index"[All Fields]) OR "bleeding on probing gingival"[All Fields]) OR "Gingival Bleeding on Probing"[All Fields]) OR "Periodontal probing depth"[All Fields]) OR "Clinical attachment loss"[All Fields]) OR "nonsurgical periodontal treatment"[All Fields]) OR "non-surgical periodontal treatment"[All Fields]) OR "loss periodontal attachment"[All Fields]) OR "attachment loss periodontal"[All Fields]) OR "periodontal attachment loss"[All Fields]) OR "clinical attachment level"[All Fields])**	
**Cochrane**	(("Diabetes Mellitus" OR " Type 2 Diabetes Mellitus" OR "Diabetes" OR "Metabolic Diseases" OR "Hyperglycemia" OR "Glucose Metabolism Disorders" OR "Metabolic Control" OR "Glycated Hemoglobin A" OR "HbA1" OR "Glycosylated Hemoglobin A" OR "Blood Glucose" OR "Glycemic Index" OR "Fasting blood glucose" OR "Post-prandial blood glucose" OR "diabetes mellitus type 2" OR "diabetes type 2" OR "type II diabetes mellitus" OR "type 2 diabetes" OR "type II diabetes" OR "advanced glycation end products" OR "HbA1c" OR "human hemoglobin A1c protein" OR "glycohemoglobin A" OR "glycosylated analysis hemoglobin A " OR "glycated hemoglobins" OR "glycated hemoglobin" OR "blood glucose metabolism" OR "blood glucose analysis") AND ("Antioxidants" OR "Antioxidant Effect" OR "Anti-Oxidant" OR "Anti Oxidant" OR "Anti-Oxidant Effects" OR "Anti Oxidant Effects" OR "Antioxidant Effects" OR "Vitamin C" OR "Vitamin-C" OR "Ascorbic acid" OR "Ascorbic-acid" OR "L-Ascorbic Acid" OR "L	1st search 124<br>2nd search 62

	Ascorbic Acid" OR "Vitamin E" OR "Vitamin-E" OR "carotenoid" OR "beta carotene" OR "beta-carotene" OR "β-carotene" OR "flavonoid" OR "flavanol" OR "flavanone" OR "catechin" OR "isoflavone" OR "selenium" OR "anthocyanidin" OR "anthocyanins" OR "tocopherols" OR "alpha-tocopherol" OR "alphatocopherol" OR "vitamin A" OR "vitamin-A" OR "retinol" OR "pro-vitamin A" OR "minerals" OR "antioxidant minerals" OR "zinc" OR "copper" OR "manganese" OR "Magnesium" OR "chromium" OR "coenzyme Q10" OR "curcumin" OR "zeaxanthin" OR "riboflavin" OR "taurine" OR "cobalamin" OR "Resveratrol" OR "Tea" OR "Green Tea" OR "Green Teas" OR "Black Tea" OR "Black Teas" OR "Vitamin B Complex" OR "Vitamin B" OR "Vitamin-B" OR "Thiamine" OR "Pantothenic Acid" OR "Niacinamide" OR "Lycopene" OR "Vitamin D" OR "Vitamin-D" OR "Aloe" OR "Fruit" OR "Seeds" OR "cranberry" OR "cranberry extract" OR "berries" OR "lutein" OR "Phenolic compounds" OR "quercetin" OR "Polyphenols" OR "coumaric acid" OR "vitamins" OR "catalase" OR "glutathione peroxidase" OR "superoxide dismutase" OR "acetylcysteine" OR "N-acetylcysteine" OR "Melatonin" OR "omega-3 fatty acid" OR "tannins") AND ("Chronic Periodontitis" OR "Chronic Periodontitides" OR "Adult Periodontitis" OR "Adult Periodontitides" OR "Periodontal treatment" OR "Periodontal therapy" OR "nonsurgical periodontal therapy" OR "non-surgical periodontal therapy" OR "Periodontitis" OR "Periodontitides" OR "Periodontal Diseases" OR "Periodontal Disease" OR "Parodontosis" OR "Parodontoses" OR "Pyorrhea Alveolaris" OR "scaling and root planing" OR "Periodontal Pocket" OR "phase 1 periodontal therapy" OR "initial periodontal therapy" OR "Periodontal Index" OR "Gingival Bleeding on Probing" OR "Periodontal probing depth" OR "Clinical attachment loss" OR "nonsurgical periodontal treatment" OR "non-surgical periodontal treatment" OR "periodontal attachment loss" OR "clinical attachment level")):ti,ab,kw" (Word variations have been searched)	
**Embase**	('diabetes mellitus'/de OR 'diabetes mellitus' OR 'type 2 diabetes mellitus'/de OR 'type 2 diabetes mellitus' OR 'diabetes'/de OR 'diabetes' OR 'metabolic diseases'/de OR 'metabolic diseases' OR 'hyperglycemia'/de OR 'hyperglycemia' OR 'glucose metabolism disorders'/de OR 'glucose metabolism disorders' OR 'metabolic control'/de OR 'metabolic control' OR 'glycated hemoglobin a'/de OR 'glycated hemoglobin a' OR 'hba1' OR 'glycosylated hemoglobin a' OR 'blood glucose'/de OR 'blood glucose' OR 'glycemic index'/de OR 'glycemic index' OR 'fasting blood glucose'/de OR 'fasting blood glucose' OR 'post-prandial blood glucose' OR 'diabetes mellitus type 2'/de OR 'diabetes mellitus type 2' OR 'diabetes type 2'/de OR 'diabetes type 2' OR 'type ii diabetes mellitus' OR 'type 2 diabetes'/de OR 'type 2 diabetes' OR 'type ii diabetes'/de OR 'type ii diabetes' OR 'advanced glycation end products' OR 'hba1c'/de OR 'hba1c' OR 'human hemoglobin a1c protein' OR 'glycohemoglobin a' OR 'glycosylated analysis hemoglobin a' OR 'glycated hemoglobins' OR 'glycated hemoglobin'/de OR 'glycated hemoglobin' OR 'blood glucose metabolism' OR 'blood glucose analysis') AND ('antioxidants'/de OR 'antioxidants' OR 'antioxidant effect' OR 'anti-oxidant' OR 'anti oxidant' OR 'anti-oxidant effects' OR 'anti oxidant effects' OR 'antioxidant effects' OR 'vitamin c'/de OR 'vitamin c' OR 'vitamin-c'/de OR 'vitamin-c' OR 'ascorbic acid'/de OR 'ascorbic acid' OR 'ascorbic-acid'/de OR 'ascorbic-acid' OR 'l-ascorbic acid'/de OR 'l-ascorbic acid' OR 'l ascorbic acid'/de OR 'l ascorbic acid' OR 'vitamin e'/de OR 'vitamin e' OR 'vitamin-e'/de OR 'vitamin-e' OR 'carotenoid'/de OR 'carotenoid' OR 'beta carotene'/de OR 'beta carotene' OR 'beta-carotene'/de OR 'beta-carotene' OR 'β-carotene' OR 'flavonoid'/de OR 'flavonoid' OR 'flavanol'/de OR 'flavanol' OR 'flavanone'/de OR 'flavanone' OR 'catechin'/de OR 'catechin' OR 'isoflavone'/de OR 'isoflavone' OR 'selenium'/de OR 'selenium' OR 'anthocyanidin'/de OR 'anthocyanidin' OR 'anthocyanins'/de OR 'anthocyanins' OR 'tocopherols'/de OR 'tocopherols' OR	1st search 441<br>2nd search 541

	'alpha-tocopherol'/de OR 'alpha-tocopherol' OR 'alphatocopherol' OR 'vitamin a'/de OR 'vitamin a' OR 'vitamin-a'/de OR 'vitamin-a' OR 'retinol'/de OR 'retinol' OR 'pro-vitamin a' OR 'minerals'/de OR 'minerals' OR 'antioxidant minerals' OR 'zinc'/de OR 'zinc' OR 'copper'/de OR 'copper' OR 'manganese'/de OR 'manganese' OR 'magnesium'/de OR 'magnesium' OR 'chromium'/de OR 'chromium' OR 'coenzyme q10'/de OR 'coenzyme q10' OR 'curcumin'/de OR 'curcumin' OR 'zeaxanthin'/de OR 'zeaxanthin' OR 'riboflavin'/de OR 'riboflavin' OR 'taurine'/de OR 'taurine' OR 'cobalamin'/de OR 'cobalamin' OR 'resveratrol'/de OR 'resveratrol' OR 'tea'/de OR 'tea' OR 'green tea'/de OR 'green tea' OR 'green teas' OR 'black tea'/de OR 'black tea' OR 'black teas' OR 'vitamin b complex'/de OR 'vitamin b complex' OR 'vitamin b'/de OR 'vitamin b' OR 'vitamin-b'/de OR 'vitamin-b' OR 'thiamine'/de OR 'thiamine' OR 'pantothenic acid'/de OR 'pantothenic acid' OR 'niacinamide'/de OR 'niacinamide' OR 'lycopene'/de OR 'lycopene' OR 'vitamin d'/de OR 'vitamin d' OR 'vitamin-d'/de OR 'vitamin-d' OR 'aloe'/de OR 'aloe' OR 'fruit'/de OR 'fruit' OR 'seeds'/de OR 'seeds' OR 'cranberry'/de OR 'cranberry' OR 'cranberry extract'/de OR 'cranberry extract' OR 'berries'/de OR 'berries' OR 'lutein'/de OR 'lutein' OR 'phenolic compounds'/de OR 'phenolic compounds' OR 'quercetin'/de OR 'quercetin' OR 'polyphenols'/de OR 'polyphenols' OR 'coumaric acid'/de OR 'coumaric acid' OR 'vitamins'/de OR 'vitamins' OR 'catalase'/de OR 'catalase' OR 'glutathione peroxidase'/de OR 'glutathione peroxidase' OR 'superoxide dismutase'/de OR 'superoxide dismutase' OR 'acetylcysteine'/de OR 'acetylcysteine' OR 'n-acetylcysteine'/de OR 'n-acetylcysteine' OR 'melatonin'/de OR 'melatonin' OR 'omega-3 fatty acid'/de OR 'omega-3 fatty acid' OR 'tannins'/de OR 'tannins') AND ('chronic periodontitis'/de OR 'chronic periodontitis' OR 'chronic periodontitides' OR 'adult periodontitis'/de OR 'adult periodontitis' OR 'adult periodontitides' OR 'periodontal treatment' OR 'periodontal therapy'/de OR 'periodontal therapy' OR 'nonsurgical periodontal therapy' OR 'non-surgical periodontal therapy' OR 'periodontitis'/de OR 'periodontitis' OR 'periodontitides' OR 'periodontal diseases'/de OR 'periodontal diseases' OR 'periodontal disease'/de OR 'periodontal disease' OR 'parodontosis'/de OR 'parodontosis' OR 'parodontoses' OR 'pyorrhea alveolaris'/de OR 'pyorrhea alveolaris' OR 'scaling and root planing'/de OR 'scaling and root planing' OR 'periodontal pocket'/de OR 'periodontal pocket' OR 'phase 1 periodontal therapy' OR 'initial periodontal therapy' OR 'periodontal index'/de OR 'periodontal index' OR 'gingival bleeding on probing' OR 'periodontal probing depth'/de OR 'periodontal probing depth' OR 'clinical attachment loss'/de OR 'clinical attachment loss' OR 'nonsurgical periodontal treatment' OR 'non-surgical periodontal treatment' OR 'periodontal attachment loss'/de OR 'periodontal attachment loss' OR 'clinical attachment level'/de OR 'clinical attachment level')	
**LIVIVO**	**("Diabetes Mellitus" OR " Type 2 Diabetes Mellitus" OR "Diabetes" OR "Metabolic Diseases" OR "Hyperglycemia" OR "Glucose Metabolism Disorders" OR "Metabolic Control" OR "Glycated Hemoglobin A" OR "HbA1" OR "Glycosylated Hemoglobin A" OR "Blood Glucose" OR "Glycemic Index" OR "Fasting blood glucose" OR "Post-prandial blood glucose"** OR "diabetes mellitus type 2" OR "diabetes type 2" OR "type II diabetes mellitus" OR "type 2 diabetes" OR "type II diabetes" OR "advanced glycation end products" OR "HbA1c" OR "human hemoglobin A1c protein" OR "glycohemoglobin A" OR "glycosylated analysis hemoglobin A " OR "glycated hemoglobins" OR "glycated hemoglobin" OR "blood glucose metabolism" OR "blood glucose analysis") **AND ("Antioxidants" OR "Antioxidant Effect" OR "Anti-Oxidant" OR "Anti Oxidant" OR "Anti-Oxidant Effects" OR "Anti Oxidant Effects" OR "Antioxidant Effects" OR "Vitamin C" OR "Vitamin-C" OR "Ascorbic acid" OR "Ascorbic-acid" OR "L-Ascorbic Acid" OR "L Ascorbic Acid" OR "Vitamin E" OR "Vitamin-E" OR "carotenoid" OR "beta**	1st search 297<br><br>2nd search 347

	**carotene" OR "beta-carotene" OR "β-carotene" OR "flavonoid" OR "flavanol" OR "flavanone" OR "catechin" OR "isoflavone" OR "selenium" OR "anthocyanidin" OR "anthocyanins" OR "tocopherols" OR "alpha-tocopherol" OR "alphatocopherol" OR "vitamin A" OR "vitamin-A" OR "retinol" OR "pro-vitamin A" OR "minerals" OR "antioxidant minerals" OR "zinc" OR "copper" OR "manganese" OR "Magnesium" OR "chromium" OR "coenzyme Q10" OR "curcumin" OR "zeaxanthin" OR "riboflavin" OR "taurine" OR "cobalamin" OR "Resveratrol" OR "Tea" OR "Green Tea" OR "Green Teas" OR "Black Tea" OR "Black Teas" OR "Vitamin B Complex" OR "Vitamin B" OR "Vitamin-B" OR "Thiamine" OR "Pantothenic Acid" OR "Niacinamide" OR "Lycopene" OR "Vitamin D" OR "Vitamin-D" OR "Aloe" OR "Fruit" OR "Seeds" OR "cranberry" OR "cranberry extract" OR "berries" OR "lutein" OR "Phenolic compounds" OR "quercetin" OR "Polyphenols" OR "coumaric acid" OR "vitamins" OR "catalase" OR "glutathione peroxidase" OR "superoxide dismutase" OR "acetylcysteine" OR "N-acetylcysteine" OR "Melatonin" OR "omega-3 fatty acid" OR "tannins")** AND **("Chronic Periodontitis" OR "Chronic Periodontitides" OR "Adult Periodontitis" OR "Adult Periodontitides" OR "Periodontal treatment" OR "Periodontal therapy" OR "nonsurgical periodontal therapy" OR "non-surgical periodontal therapy" OR "Periodontitis" OR "Periodontitides" OR "Periodontal Diseases" OR "Periodontal Disease" OR "Parodontosis" OR "Parodontoses" OR "Pyorrhea Alveolaris" OR "scaling and root planing" OR "Periodontal Pocket" OR "phase 1 periodontal therapy" OR "initial periodontal therapy" OR "Periodontal Index" OR "Gingival Bleeding on Probing" OR "Periodontal probing depth" OR "Clinical attachment loss" OR "nonsurgical periodontal treatment" OR "non-surgical periodontal treatment" OR "periodontal attachment loss" OR "clinical attachment level")**	
**Scopus**	**TITLE-ABS-KEY (("Diabetes Mellitus" OR " Type 2 Diabetes Mellitus" OR "Diabetes" OR "Metabolic Diseases" OR "Hyperglycemia" OR "Glucose Metabolism Disorders" OR "Metabolic Control" OR "Glycated Hemoglobin A" OR "HbA1" OR "Glycosylated Hemoglobin A" OR "Blood Glucose" OR "Glycemic Index" OR "Fasting blood glucose" OR "Post-prandial blood glucose" OR "diabetes mellitus type 2" OR "diabetes type 2" OR "type II diabetes mellitus" OR "type 2 diabetes" OR "type II diabetes" OR "advanced glycation end products" OR "HbA1c" OR "human hemoglobin A1c protein" OR "glycohemoglobin A" OR "glycosylated analysis hemoglobin A " OR "glycated hemoglobins" OR "glycated hemoglobin" OR "blood glucose metabolism" OR "blood glucose analysis") AND ("Antioxidants" OR "Antioxidant Effect" OR "Anti-Oxidant" OR "Anti Oxidant" OR "Anti-Oxidant Effects" OR "Anti Oxidant Effects" OR "Antioxidant Effects" OR "Vitamin C" OR "Vitamin-C" OR "Ascorbic acid" OR "Ascorbic-acid" OR "L-Ascorbic Acid" OR "L Ascorbic Acid" OR "Vitamin E" OR "Vitamin-E" OR "carotenoid" OR "beta carotene" OR "beta-carotene" OR "β-carotene" OR "flavonoid" OR "flavanol" OR "flavanone" OR "catechin" OR "isoflavone" OR "selenium" OR "anthocyanidin" OR "anthocyanins" OR "tocopherols" OR "alpha-tocopherol" OR "alphatocopherol" OR "vitamin A" OR "vitamin-A" OR "retinol" OR "pro-vitamin A" OR "minerals" OR "antioxidant minerals" OR "zinc" OR "copper" OR "manganese" OR "Magnesium" OR "chromium" OR "coenzyme Q10" OR "curcumin" OR "zeaxanthin" OR "riboflavin" OR "taurine" OR "cobalamin" OR "Resveratrol" OR "Tea" OR "Green Tea" OR "Green Teas" OR "Black Tea" OR "Black Teas" OR "Vitamin B Complex" OR "Vitamin B" OR "Vitamin-B" OR "Thiamine" OR "Pantothenic Acid" OR "Niacinamide" OR "Lycopene" OR "Vitamin D" OR "Vitamin-D" OR "Aloe" OR "Fruit" OR "Seeds" OR "cranberry" OR "cranberry extract" OR "berries" OR "lutein" OR "Phenolic compounds" OR "quercetin" OR "Polyphenols" OR "coumaric acid" OR "vitamins" OR**	1st search 474<br><br>2nd search 578

	**"catalase" OR "glutathione peroxidase" OR "superoxide dismutase" OR "acetylcysteine" OR "N-acetylcysteine" OR "Melatonin" OR "omega-3 fatty acid" OR "tannins") AND ("Chronic Periodontitis" OR "Chronic Periodontitides" OR "Adult Periodontitis" OR "Adult Periodontitides" OR "Periodontal treatment" OR "Periodontal therapy" OR "nonsurgical periodontal therapy" OR "non-surgical periodontal therapy" OR "Periodontitis" OR "Periodontitides" OR "Periodontal Diseases" OR "Periodontal Disease" OR "Parodontosis" OR "Parodontoses" OR "Pyorrhea Alveolaris" OR "scaling and root planing" OR "Periodontal Pocket" OR "phase 1 periodontal therapy" OR "initial periodontal therapy" OR "Periodontal Index" OR "Gingival Bleeding on Probing" OR "Periodontal probing depth" OR "Clinical attachment loss" OR "nonsurgical periodontal treatment" OR "non-surgical periodontal treatment" OR "periodontal attachment loss" OR "clinical attachment level"))**	
**Web of Science**	**(("Diabetes Mellitus" OR " Type 2 Diabetes Mellitus" OR "Diabetes" OR "Metabolic Diseases" OR "Hyperglycemia" OR "Glucose Metabolism Disorders" OR "Metabolic Control" OR "Glycated Hemoglobin A" OR "HbA1" OR "Glycosylated Hemoglobin A" OR "Blood Glucose" OR "Glycemic Index" OR "Fasting blood glucose" OR "Post-prandial blood glucose" OR "diabetes mellitus type 2" OR "diabetes type 2" OR "type II diabetes mellitus" OR "type 2 diabetes" OR "type II diabetes" OR "advanced glycation end products" OR "HbA1c" OR "human hemoglobin A1c protein" OR "glycohemoglobin A" OR "glycosylated analysis hemoglobin A " OR "glycated hemoglobins" OR "glycated hemoglobin" OR "blood glucose metabolism" OR "blood glucose analysis") AND ("Antioxidants" OR "Antioxidant Effect" OR "Anti-Oxidant" OR "Anti Oxidant" OR "Anti-Oxidant Effects" OR "Anti Oxidant Effects" OR "Antioxidant Effects" OR "Vitamin C" OR "Vitamin-C" OR "Ascorbic acid" OR "Ascorbic-acid" OR "L-Ascorbic Acid" OR "L Ascorbic Acid" OR "Vitamin E" OR "Vitamin-E" OR "carotenoid" OR "beta carotene" OR "beta-carotene" OR "β-carotene" OR "flavonoid" OR "flavanol" OR "flavanone" OR "catechin" OR "isoflavone" OR "selenium" OR "anthocyanidin" OR "anthocyanins" OR "tocopherols" OR "alpha-tocopherol" OR "alphatocopherol" OR "vitamin A" OR "vitamin-A" OR "retinol" OR "pro-vitamin A" OR "minerals" OR "antioxidant minerals" OR "zinc" OR "copper" OR "manganese" OR "Magnesium" OR "chromium" OR "coenzyme Q10" OR "curcumin" OR "zeaxanthin" OR "riboflavin" OR "taurine" OR "cobalamin" OR "Resveratrol" OR "Tea" OR "Green Tea" OR "Green Teas" OR "Black Tea" OR "Black Teas" OR "Vitamin B Complex" OR "Vitamin B" OR "Vitamin-B" OR "Thiamine" OR "Pantothenic Acid" OR "Niacinamide" OR "Lycopene" OR "Vitamin D" OR "Vitamin-D" OR "Aloe" OR "Fruit" OR "Seeds" OR "cranberry" OR "cranberry extract" OR "berries" OR "lutein" OR "Phenolic compounds" OR "quercetin" OR "Polyphenols" OR "coumaric acid" OR "vitamins" OR "catalase" OR "glutathione peroxidase" OR "superoxide dismutase" OR "acetylcysteine" OR "N-acetylcysteine" OR "Melatonin" OR "omega-3 fatty acid" OR "tannins") AND ("Chronic Periodontitis" OR "Chronic Periodontitides" OR "Adult Periodontitis" OR "Adult Periodontitides" OR "Periodontal treatment" OR "Periodontal therapy" OR "nonsurgical periodontal therapy" OR "non-surgical periodontal therapy" OR "Periodontitis" OR "Periodontitides" OR "Periodontal Diseases" OR "Periodontal Disease" OR "Parodontosis" OR "Parodontoses" OR "Pyorrhea Alveolaris" OR "scaling and root planing" OR "Periodontal Pocket" OR "phase 1 periodontal therapy" OR "initial periodontal therapy" OR "Periodontal Index" OR "Gingival Bleeding on Probing" OR "Periodontal probing depth" OR "Clinical attachment loss" OR "nonsurgical periodontal treatment" OR "non-surgical periodontal treatment" OR "periodontal attachment loss" OR "clinical attachment level"))<br>Tempo estipulado: Todos os anos. Índices: SCI-EXPANDED, SSCI, A&HCI, CPCI-S, CPCI-SSH, ESCI.**	1st search 203<br><br>2nd search 239

**LILACS (Portuguese and Spanish)**	(tw:(("Diabetes Mellitus" OR " Type 2 Diabetes Mellitus" OR "Diabetes" OR "Metabolic Diseases" OR "Hyperglycemia" OR "Glucose Metabolism Disorders" OR "Metabolic Control" OR "Glycated Hemoglobin A" OR "HbA1" OR "Glycosylated Hemoglobin A" OR "Blood Glucose" OR "Glycemic Index" OR "Fasting blood glucose" OR "Post-prandial blood glucose" OR "diabetes mellitus type 2" OR "diabetes type 2" OR "type II diabetes mellitus" OR "type 2 diabetes" OR "type II diabetes" OR "advanced glycation end products" OR "HbA1c" OR "human hemoglobin A1c protein" OR "glycohemoglobin A" OR "glycosylated analysis hemoglobin A " OR "glycated hemoglobins" OR "glycated hemoglobin" OR "blood glucose metabolism" OR "blood glucose analysis" OR "Diabetes Mellitus" OR "Diabetes mellitus tipo 2" OR "doenças metabólicas" OR "enfermedades metabólicas" OR "Hiperglicemia" OR "hiperglucemia" OR "Tanstornos do metabolismo de glucose" OR "Transtornos del metabolismo de la glucosa" OR "Hemoglobina A Glicada" OR "Hemoglobina A Glucada" OR "glicemia" OR "glucemia" OR "índice glicêmico" OR "índice glucémico" OR "produtos finais de glicação avançada" OR "productos finales de glicación avanzada") AND ("Antioxidants" OR "Antioxidant Effect" OR "Anti-Oxidant" OR "Anti Oxidant" OR "Anti-Oxidant Effects" OR "Anti Oxidant Effects" OR "Antioxidant Effects" OR "Vitamin C" OR "Vitamin-C" OR "Ascorbic acid" OR "Ascorbic-acid" OR "L-Ascorbic Acid" OR "L Ascorbic Acid" OR "Vitamin E" OR "Vitamin-E" OR "carotenoid" OR "beta carotene" OR "beta-carotene" OR "β-carotene" OR "flavonoid" OR "flavanol" OR "flavanone" OR "catechin" OR "isoflavone" OR "selenium" OR "anthocyanidin" OR "anthocyanins" OR "tocopherols" OR "alpha-tocopherol" OR "alphatocopherol" OR "vitamin A" OR "vitamin-A" OR "retinol" OR "pro-vitamin A" OR "minerals" OR "antioxidant minerals" OR "zinc" OR "copper" OR "manganese" OR "Magnesium" OR "chromium" OR "coenzyme Q10" OR "curcumin" OR "zeaxanthin" OR "riboflavin" OR "taurine" OR "cobalamin" OR "Resveratrol" OR "Tea" OR "Green Tea" OR "Green Teas" OR "Black Tea" OR "Black Teas" OR "Vitamin B Complex" OR "Vitamin B" OR "Vitamin-B" OR "Thiamine" OR "Pantothenic Acid" OR "Niacinamide" OR "Lycopene" OR "Vitamin D" OR "Vitamin-D" OR "Aloe" OR "Fruit" OR "Seeds" OR "cranberry" OR "cranberry extract" OR "berries" OR "lutein" OR "Phenolic compounds" OR "quercetin" OR "Polyphenols" OR "coumaric acid" OR "vitamins" OR "catalase" OR "glutathione peroxidase" OR "superoxide dismutase" OR "acetylcysteine" OR "N-acetylcysteine" OR "Melatonin" OR "omega-3 fatty acid" OR "tannins" OR "Antioxidantes" OR "Ácido ascórbico" OR "Vitamina E" OR "beta caroteno" OR "catequina" OR "isoflavonas" OR "selênio" OR "selenio" OR "antocianinas" OR "tocoferóis" OR "tocoferoles" OR "vitamina A" OR "minerais" OR "minerales" OR "zinco" OR "zinc" OR "cobre" OR "manganês" OR "manganeso" OR "magnésio" OR "magnesio" OR "cromo" OR "ubiquinona" OR "curcumina" OR "zeaxantinas" OR "riboflavina" OR "taurina" OR "vitamina B 12" OR "Resveratrol" OR "Chá" OR "Té" OR "complexo vitamínico B" OR "complejo vitamínico B" OR "tiamina" OR "ácido pantotênico" OR "ácido pantoténico" OR "niacinamida" OR "licopeno" OR "vitamina D" OR "Aloe" OR "Frutas" OR "Sementes" OR "Semillas" OR "Vaccinium macrocarpon" OR "luteína" OR "compostos fenólicos" OR "compuestos fenólicos" OR "quercetina" OR "Polifenóis" OR "polifenoles" OR "ácidos cumáricos" OR "vitaminas" OR "catalase" OR "catalasa" OR "glutationa peroxidase" OR "glutatión peroxidasa" OR "superóxido dismutase" OR "superóxido dismutasa" OR "acetilcisteína" OR "melatonina" OR "ácidos graxos ômega-3" OR "ácidos grasos omega-3" OR "taninos") AND ("Chronic Periodontitis" OR "Chronic Periodontitides" OR "Adult Periodontitis" OR "Adult Periodontitides" OR "Periodontal treatment" OR "Periodontal therapy" OR "nonsurgical periodontal therapy" OR "non-surgical periodontal therapy" OR "Periodontitis" OR "Periodontitides" OR "Periodontal Diseases" OR "Periodontal Disease" OR "Parodontosis" OR "Parodontoses" OR "Pyorrhea Alveolaris" OR "scaling and root planing" OR "Periodontal Pocket" OR "phase 1 periodontal therapy" OR "initial	1st search 11<br><br>2nd search 13

	periodontal therapy" OR "Periodontal Index" OR "Gingival Bleeding on Probing" OR "Periodontal probing depth" OR "Clinical attachment loss" OR "nonsurgical periodontal treatment" OR "non-surgical periodontal treatment" OR "periodontal attachment loss" OR "clinical attachment level" OR "Periodontite crônica" OR "periodontitis crónica" OR "desbridamento periodontal" OR "aplainamento radicular" OR "aplanamiento de la raíz" OR "raspagem dentária" OR "raspado dental" OR "curetagem subgengival" OR "curetaje subgingival" OR "Periodontite" OR "Periodontitis" OR "doenças periodontais" OR "enfermedades periodontales" OR "bolsa periodontal" OR "índice periodontal" OR "perda da inserção periodontal" OR "pérdida de la inserción periodontal")))	
**ProQuest**	**noft(("Diabetes Mellitus" OR " Type 2 Diabetes Mellitus" OR "Diabetes" OR "Metabolic Diseases" OR "Hyperglycemia" OR "Glucose Metabolism Disorders" OR "Metabolic Control" OR "Glycated Hemoglobin A" OR "HbA1" OR "Glycosylated Hemoglobin A" OR "Blood Glucose" OR "Glycemic Index" OR "Fasting blood glucose" OR "Post-prandial blood glucose" OR "diabetes mellitus type 2" OR "diabetes type 2" OR "type II diabetes mellitus" OR "type 2 diabetes" OR "type II diabetes" OR "advanced glycation end products" OR "HbA1c" OR "human hemoglobin A1c protein" OR "glycohemoglobin A" OR "glycosylated analysis hemoglobin A " OR "glycated hemoglobins" OR "glycated hemoglobin" OR "blood glucose metabolism" OR "blood glucose analysis") AND ("Antioxidants" OR "Antioxidant Effect" OR "Anti-Oxidant" OR "Anti Oxidant" OR "Anti-Oxidant Effects" OR "Anti Oxidant Effects" OR "Antioxidant Effects" OR "Vitamin C" OR "Vitamin-C" OR "Ascorbic acid" OR "Ascorbic-acid" OR "L-Ascorbic Acid" OR "L Ascorbic Acid" OR "Vitamin E" OR "Vitamin-E" OR "carotenoid" OR "beta carotene" OR "beta-carotene" OR "β-carotene" OR "flavonoid" OR "flavanol" OR "flavanone" OR "catechin" OR "isoflavone" OR "selenium" OR "anthocyanidin" OR "anthocyanins" OR "tocopherols" OR "alpha-tocopherol" OR "alphatocopherol" OR "vitamin A" OR "vitamin-A" OR "retinol" OR "pro-vitamin A" OR "minerals" OR "antioxidant minerals" OR "zinc" OR "copper" OR "manganese" OR "Magnesium" OR "chromium" OR "coenzyme Q10" OR "curcumin" OR "zeaxanthin" OR "riboflavin" OR "taurine" OR "cobalamin" OR "Resveratrol" OR "Tea" OR "Green Tea" OR "Green Teas" OR "Black Tea" OR "Black Teas" OR "Vitamin B Complex" OR "Vitamin B" OR "Vitamin-B" OR "Thiamine" OR "Pantothenic Acid" OR "Niacinamide" OR "Lycopene" OR "Vitamin D" OR "Vitamin-D" OR "Aloe" OR "Fruit" OR "Seeds" OR "cranberry" OR "cranberry extract" OR "berries" OR "lutein" OR "Phenolic compounds" OR "quercetin" OR "Polyphenols" OR "coumaric acid" OR "vitamins" OR "catalase" OR "glutathione peroxidase" OR "superoxide dismutase" OR "acetylcysteine" OR "N-acetylcysteine" OR "Melatonin" OR "omega-3 fatty acid" OR "tannins") AND ("Chronic Periodontitis" OR "Chronic Periodontitides" OR "Adult Periodontitis" OR "Adult Periodontitides" OR "Periodontal treatment" OR "Periodontal therapy" OR "nonsurgical periodontal therapy" OR "non-surgical periodontal therapy" OR "Periodontitis" OR "Periodontitides" OR "Periodontal Diseases" OR "Periodontal Disease" OR "Parodontosis" OR "Parodontoses" OR "Pyorrhea Alveolaris" OR "scaling and root planing" OR "Periodontal Pocket" OR "phase 1 periodontal therapy" OR "initial periodontal therapy" OR "Periodontal Index" OR "Gingival Bleeding on Probing" OR "Periodontal probing depth" OR "Clinical attachment loss" OR "nonsurgical periodontal treatment" OR "non-surgical periodontal treatment" OR "periodontal attachment loss" OR "clinical attachment level"))**	1st search 8<br>2nd search 12
**Open Grey**	**("Diabetes Mellitus" OR " Type 2 Diabetes Mellitus" OR "Diabetes" OR "Metabolic Diseases" OR "Hyperglycemia" OR "Glucose Metabolism Disorders" OR "Metabolic Control" OR "Glycated Hemoglobin A" OR "HbA1" OR "Glycosylated Hemoglobin A" OR "Blood Glucose" OR "Glycemic Index" OR "Fasting blood glucose" OR "Post-prandial blood glucose"** OR "diabetes mellitus type 2" OR "diabetes type 2" OR "type II diabetes mellitus" OR "type 2 diabetes" OR "type II diabetes" OR "advanced glycation end products" OR "HbA1c" OR "human hemoglobin A1c protein" OR "glycohemoglobin A" OR "glycosylated analysis	1st search 0<br>2nd search 0

	hemoglobin A " OR "glycated hemoglobins" OR "glycated hemoglobin" OR "blood glucose metabolism" OR "blood glucose analysis") **AND ("Antioxidants" OR "Antioxidant Effect" OR "Anti-Oxidant" OR "Anti Oxidant" OR "Anti-Oxidant Effects" OR "Anti Oxidant Effects" OR "Antioxidant Effects" OR "Vitamin C" OR "Vitamin-C" OR "Ascorbic acid" OR "Ascorbic-acid" OR "L-Ascorbic Acid" OR "L Ascorbic Acid" OR "Vitamin E" OR "Vitamin-E" OR "carotenoid" OR "beta carotene" OR "beta-carotene" OR "β-carotene" OR "flavonoid" OR "flavanol" OR "flavanone" OR "catechin" OR "isoflavone" OR "selenium" OR "anthocyanidin" OR "anthocyanins" OR "tocopherols" OR "alpha-tocopherol" OR "alphatocopherol" OR "vitamin A" OR "vitamin-A" OR "retinol" OR "pro-vitamin A" OR "minerals" OR "antioxidant minerals" OR "zinc" OR "copper" OR "manganese" OR "Magnesium" OR "chromium" OR "coenzyme Q10" OR "curcumin" OR "zeaxanthin" OR "riboflavin" OR "taurine" OR "cobalamin" OR "Resveratrol" OR "Tea" OR "Green Tea" OR "Green Teas" OR "Black Tea" OR "Black Teas" OR "Vitamin B Complex" OR "Vitamin B" OR "Vitamin-B" OR "Thiamine" OR "Pantothenic Acid" OR "Niacinamide" OR "Lycopene" OR "Vitamin D" OR "Vitamin-D" OR "Aloe" OR "Fruit" OR "Seeds" OR "cranberry" OR "cranberry extract" OR "berries" OR "lutein" OR "Phenolic compounds" OR "quercetin" OR "Polyphenols" OR "coumaric acid" OR "vitamins" OR "catalase" OR "glutathione peroxidase" OR "superoxide dismutase" OR "acetylcysteine" OR "N-acetylcysteine" OR "Melatonin" OR "omega-3 fatty acid" OR "tannins")** AND **("Chronic Periodontitis" OR "Chronic Periodontitides" OR "Adult Periodontitis" OR "Adult Periodontitides" OR "Periodontal treatment" OR "Periodontal therapy" OR "nonsurgical periodontal therapy" OR "non-surgical periodontal therapy" OR "Periodontitis" OR "Periodontitides" OR "Periodontal Diseases" OR "Periodontal Disease" OR "Parodontosis" OR "Parodontoses" OR "Pyorrhea Alveolaris" OR "scaling and root planing" OR "Periodontal Pocket" OR "phase 1 periodontal therapy" OR "initial periodontal therapy" OR "Periodontal Index" OR "Gingival Bleeding on Probing" OR "Periodontal probing depth" OR "Clinical attachment loss" OR "nonsurgical periodontal treatment" OR "non-surgical periodontal treatment" OR "periodontal attachment loss" OR "clinical attachment level")**	
**Google Scholar**	("diabetes mellitus type 2" OR "diabetes mellitus") AND (periodontitis OR "Chronic Periodontitis") AND ("non-surgical periodontal therapy" OR "scaling and root planing") AND ("Antioxidants" OR "Anti-Oxidant") AND ("glycosylated hemoglobin" OR "HbA1")	1st search 37<br><br>2nd search 47
**Clinical Trial registry**	Condition or disease: Periodontitis<br>Other terms: Antioxidants AND diabetes	1st search Not performed<br><br>2nd search 8

Supplementary File S2: Excluded articles and reason for exclusion. (n=14)

Author, year	Reason for exclusion
Babaei et al., 2018	7
Bazyar et al., 2018	6
Castro dos Santos et al., 2020	9
Elwakeel et al., 2015	9
Gokhale et al., 2013	6
Gorbacheva et al., 2010	10
Özcengiz, 2006	5
Zare Javid et al., 2016a	7
Zare Javid et al., 2016b	7
Zare Javid et al., 2017	5
Zare Javid et al., 2019a	6
Zare Javid et al., 2019b	6
Zare Javid et al., 2020	6
Zorina et al., 2017	8

1- Reviews, letters, personal opinions, books or book chapters, panel and conference abstracts (n= 0), 2- Wrong design studies. (n= 0), 3- Wrong sample studies. (n=0), 4- Study Protocols (n= 0), 5- Studies written in non-Latin alphabets (n=2), 6- Wrong outcome (n= 5), 7- Wrong population (n=3), 8- Wrong drug (n=1), 9- Confused effects (n=2), 10- No full text available (n=1).

REFERENCES

1. Babaei H, Forouzandeh F, Maghsoumi-Norouzabad L, Yousefimanesh HA, Ravanbakhsh M, Zare Javid A. Effects of Chicory Leaf Extract on Serum Oxidative Stress Markers, Lipid Profile and Periodontal Status in Patients With Chronic Periodontitis. J Am Coll Nutr. 2018; 37(6):479-486
2. Bazyar H, Gholinezhad H, Moradi L, Salehi P, Abadi F, Ravanbakhsh M, Zare Javid A. The effects of melatonin supplementation in adjunct with non-surgical periodontal therapy on periodontal status, serum melatonin and inflammatory markers in type 2 diabetes mellitus patients with chronic periodontitis: a double-blind, placebo-controlled trial. Inflammopharmacology. 2018; 27(1):67-76.
3. Castro Dos Santos NC, Andere NMRB, Araujo CF, de Marco AC, Kantarci A, Van Dyke TE, Santamaria MP. Omega-3 PUFA and aspirin as adjuncts to periodontal debridement in patients with periodontitis and type 2 diabetes mellitus: Randomized clinical trial. J Periodontol. 2020; 19-0613.

4. Elwakeel NM, Hazaa HH. Effect of omega 3 fatty acids plus low-dose aspirin on both clinical and biochemical profiles of patients with chronic periodontitis and type 2 diabetes: a randomized double blind placebo-controlled study. J Periodontal Res. 2015; 50:721-729.
5. Gokhale NH, Acharya AB, Patil VS, Trivedi DJ, Thakur SL. A short-term evaluation of the relationship between plasma ascorbic acid levels and periodontal disease in systemically healthy and type 2 diabetes mellitus subjects. J Diet Suppl. 2013;10(2):93-104.
6. Gorbacheva IA, Orekhova LY, Shestakova LA, Musayeva RS, Silina AS. Alternative pathogenetic approach to the treatment of periodontal inflammatory diseases in diabetic patients with polyorganic disorders. Azerbaijan Medical Journal. 2010; 0:81-87.
7. Özcengiz, M. Tip 2 Diabetes Mellitus'Lu Hastalarda Periodontal Tedavi Ve Antioksidan kullanımının Metabolik Kontrol üzerine Etkisi. 2006. 160. (Order No. 28521788) - Marmara Universitesi (Turkey), Ann Arbor, 2006.
8. Zare Javid A, Hormoznejad R, Yousefimanesh HA, Zakerkish M, Haghighi-Zadeh MH, Dehghan P, Ravanbakhsh M. The Impact of Resveratrol Supplementation on Blood Glucose, Insulin, Insulin Resistance, Triglyceride, and Periodontal Markers in Type 2 Diabetic Patients with Chronic Periodontitis. Phytother Res. 2016; 31(1):108-114.
9. Zare Javid A, Hormoznejad R, Yousefimanesh HA, Zakerkish M, Haghighi-zadeh MH, Barzegar A, Niknejad N. The Effect of Resveratrol Supplementation in Adjunct with Non-surgical Periodontal Treatment on Blood Glucose, Triglyceride, Periodontal Status and Some Inflammatory Markers in Type 2 Diabetic Patients with Periodontal Disease. Nutrition and Food Sciences Research. 2016; 3(1):17-26.
10. Zare Javid A, Ashrafzadeh E, Babaee H, Yousefimanesh H, Zakerkish M, Ahmadi-Angali K, Asgari G. Impact of cranberry juice enriched with omega-3 and their individual consumption adjunct with nonsurgical periodontal treatment on glycemic control and lipid profile in patients with diabetes mellitus and periodontal disease. Journal of Isfahan Medical School. 2017; 35. 919-926.
11. Zare Javid A, Hormoznejad R, Yousefimanesh HA, Haghighi-Zadeh MH, Zakerkish M. Impact of resveratrol supplementation on inflammatory, antioxidant, and periodontal markers in type 2 diabetic patients with chronic periodontitis. Diabetes Metab Syndr. 2019; 13(4):2769-2774.
12. Zare Javid A, Bazyar H, Gholinezhad H, Rahimlou M, Rashidi H, Salehi P, Haghighi-Zadeh MH. The effects of ginger supplementation on inflammatory, antioxidant, and periodontal parameters in type 2 diabetes mellitus patients with chronic periodontitis under non-surgical periodontal therapy. A double-blind, placebo-controlled trial. Diabetes Metab Syndr Obes. 2019; 12:1751-1761.
13. Zare Javid A, Hosseini SA, Gholinezhad H, Moradi L, Haghighi-Zadeh MH, Bazyar H. Antioxidant and Anti-Inflammatory Properties of Melatonin in Patients with Type 2 Diabetes Mellitus with Periodontal Disease Under Non-Surgical Periodontal Therapy: A Double-Blind, Placebo-Controlled Trial. Diabetes Metab Syndr Obes. 2020; 13:753-761.
14. Zorina OA, Petrukhina NB, Kartysheva EV, Saltovets MV. The effectiveness of com bined treatment with simbiotiks and antioxidants in patients with periodontal disease and metabolic syndrome. Vopr Pitan. 2017; 86(1):87-92.

Supplementary File S3: League table showing the effect estimates and 95%CI for each comparison. The lower triangle shows the results of mixed comparisons; the upper triangle shows direct comparisons. Negative results favor treatments showed in the columns, positive results favor treatment in the rows. Bold results indicate that the treatment in the column was more effective compared to the intervention in the line. The colors show the certainty of evidence for each comparison, according to the legend on the lower left (green: high certainty; yellow: moderate certainty; orange: low certainty; light red: very low certainty).

**Alpha Lipoic Acid**	.	.	.	.	.	.	.	.	.	**-2.43 [-3.26; -1.59]**
-0.79 [-1.84; 0.27]	**Melatonin**	.	.	.	.	.	.	.	.	**-1.64 [-2.29; -0.99]**
**-1.60 [-2.61; -0.58]**	-0.81 [-1.68; 0.06]	**Propolis**	.	.	.	.	.	.	.	**-0.83 [-1.41; -0.25]**
**-1.90 [-2.92; -0.89]**	**-1.12 [-1.98; -0.25]**	-0.31 [-1.12; 0.51]	**Grape Seeds**	.	.	.	.	.	.	-0.52 [-1.10; 0.05]
**-1.94 [-2.95; -0.93]**	**-1.15 [-2.01; -0.29]**	-0.34 [-1.15; 0.47]	-0.03 [-0.84; 0.77]	**Omega 3**	.	.	-0.44 [-1.33; 0.45]	-0.58 [-1.51; 0.34]	.	-0.49 [-1.05; 0.08]
**-1.97 [-3.01; -0.92]**	**-1.18 [-2.08; -0.28]**	-0.37 [-1.23; 0.49]	-0.06 [-0.92; 0.79]	-0.03 [-0.87; 0.82]	**Lycopene**	.	.	.	.	-0.46 [-1.09; 0.17]
**-2.10 [-3.13; -1.06]**	**-1.31 [-2.20; -0.42]**	-0.50 [-1.34; 0.34]	-0.19 [-1.03; 0.65]	-0.16 [-0.99; 0.68]	-0.13 [-1.00; 0.75]	**Ginger**	.	.	.	-0.33 [-0.94; 0.28]
**-2.35 [-3.50; -1.20]**	**-1.56 [-2.58; -0.54]**	-0.75 [-1.73; 0.23]	-0.44 [-1.42; 0.53]	-0.41 [-1.22; 0.40]	-0.38 [-1.39; 0.63]	-0.25 [-1.25; 0.74]	**Cranb + Omega 3**	-0.14 [-1.04; 0.76]	.	-0.10 [-0.94; 0.74]
**-2.49 [-3.66; -1.32]**	**-1.70 [-2.75; -0.66]**	-0.89 [-1.89; 0.11]	-0.58 [-1.59; 0.42]	-0.55 [-1.40; 0.30]	-0.52 [-1.55; 0.51]	-0.39 [-1.41; 0.63]	-0.14 [-1.04; 0.76]	**Cranberry**	.	0.04 [-0.83; 0.90]
**-2.50 [-3.59; -1.40]**	**-1.71 [-2.67; -0.75]**	-0.90 [-1.81; 0.01]	-0.59 [-1.50; 0.32]	-0.56 [-1.46; 0.35]	-0.53 [-1.47; 0.42]	-0.40 [-1.33; 0.53]	-0.15 [-1.21; 0.91]	-0.01 [-1.09; 1.07]	**Vitamin C**	0.07 [-0.63; 0.78]
**-2.43 [-3.26; -1.59]**	**-1.64 [-2.29; -0.99]**	**-0.83 [-1.41; -0.25]**	-0.52 [-1.10; 0.05]	-0.49 [-1.05; 0.08]	-0.46 [-1.09; 0.17]	-0.33 [-0.94; 0.28]	-0.08 [-0.87; 0.71]	0.06 [-0.76; 0.88]	0.07 [-0.63; 0.78]	**NSPT**

**Certainty**
High
Moderate
Low
Very Low

Supplementary File S4: Assessment of inconsistency for all studies (SMD)

Comparison	N. Studies	NMA	Direct	Indirect	Difference	Diff_95CI_lower	Diff_95CI_upper	pValue
ALA:Cranb	0	-2.49	NA	-2.49	NA	NA	NA	NA
ALA:CranbOm3	0	-2.35	NA	-2.35	NA	NA	NA	NA
ALA:Ginger	0	-2.10	NA	-2.10	NA	NA	NA	NA
ALA:GrapeS	0	-1.90	NA	-1.90	NA	NA	NA	NA
ALA:Lycop	0	-1.97	NA	-1.97	NA	NA	NA	NA
ALA:Melato	0	-0.79	NA	-0.79	NA	NA	NA	NA
ALA:NSPT	1	-2.43	-2.43	NA	NA	NA	NA	NA
ALA:Omega3	0	-1.94	NA	-1.94	NA	NA	NA	NA
ALA:Propo	0	-1.60	NA	-1.60	NA	NA	NA	NA
ALA:VitC	0	-2.50	NA	-2.50	NA	NA	NA	NA
Cranb:CranbOm3	1	0.14	0.14	NA	NA	NA	NA	NA
Cranb:Ginger	0	0.39	NA	0.39	NA	NA	NA	NA
Cranb:GrapeS	0	0.58	NA	0.58	NA	NA	NA	NA
Cranb:Lycop	0	0.52	NA	0.52	NA	NA	NA	NA
Cranb:Melato	0	1.70	NA	1.70	NA	NA	NA	NA
Cranb:NSPT	1	0.06	0.04	0.28	-0.24	-2.95	2.46	0.86
Cranb:Omega3	1	0.55	0.58	0.37	0.21	-2.10	2.52	0.86
Cranb:Propo	0	0.89	NA	0.89	NA	NA	NA	NA
Cranb:VitC	0	-0.01	NA	-0.01	NA	NA	NA	NA
CranbOm3:Ginger	0	0.25	NA	0.25	NA	NA	NA	NA
CranbOm3:GrapeS	0	0.44	NA	0.44	NA	NA	NA	NA
CranbOm3:Lycop	0	0.38	NA	0.38	NA	NA	NA	NA
CranbOm3:Melato	0	1.56	NA	1.56	NA	NA	NA	NA
CranbOm3:NSPT	1	-0.08	-0.10	0.13	-0.23	-2.71	2.24	0.85
CranbOm3:Omega3	1	0.41	0.44	0.24	0.20	-1.99	2.40	0.85
CranbOm3:Propo	0	0.75	NA	0.75	NA	NA	NA	NA
CranbOm3:VitC	0	-0.15	NA	-0.15	NA	NA	NA	NA
Ginger:GrapeS	0	0.19	NA	0.19	NA	NA	NA	NA
Ginger:Lycop	0	0.13	NA	0.13	NA	NA	NA	NA
Ginger:Melato	0	1.31	NA	1.31	NA	NA	NA	NA
Ginger:NSPT	1	-0.33	-0.33	NA	NA	NA	NA	NA
Ginger:Omega3	0	0.16	NA	0.16	NA	NA	NA	NA
Ginger:Propo	0	0.50	NA	0.50	NA	NA	NA	NA
Ginger:VitC	0	-0.40	NA	-0.40	NA	NA	NA	NA
GrapeS:Lycop	0	-0.06	NA	-0.06	NA	NA	NA	NA
GrapeS:Melato	0	1.12	NA	1.12	NA	NA	NA	NA
GrapeS:NSPT	1	-0.52	-0.52	NA	NA	NA	NA	NA
GrapeS:Omega3	0	-0.03	NA	-0.03	NA	NA	NA	NA
GrapeS:Propo	0	0.31	NA	0.31	NA	NA	NA	NA
GrapeS:VitC	0	-0.59	NA	-0.59	NA	NA	NA	NA
Lycop:Melato	0	1.18	NA	1.18	NA	NA	NA	NA

Comparison	N. Studies	NMA	Direct	Indirect	Difference	Diff_95CI_lower	Diff_95CI_upper	pValue
Lycop:NSPT	1	-0.46	-0.46	NA	NA	NA	NA	NA
Lycop:Omega3	0	0.03	NA	0.03	NA	NA	NA	NA
Lycop:Propo	0	0.37	NA	0.37	NA	NA	NA	NA
Lycop:VitC	0	-0.53	NA	-0.53	NA	NA	NA	NA
Melato:NSPT	1	-1.64	-1.64	NA	NA	NA	NA	NA
Melato:Omega3	0	-1.15	NA	-1.15	NA	NA	NA	NA
Melato:Propo	0	-0.81	NA	-0.81	NA	NA	NA	NA
Melato:VitC	0	-1.71	NA	-1.71	NA	NA	NA	NA
Omega3:NSPT	2	-0.49	-0.49	NA	NA	NA	NA	NA
Propo:NSPT	1	-0.83	-0.83	NA	NA	NA	NA	NA
VitC:NSPT	1	0.07	0.07	NA	NA	NA	NA	NA
Omega3:Propo	0	0.34	NA	0.34	NA	NA	NA	NA
Omega3:VitC	0	-0.56	NA	-0.56	NA	NA	NA	NA
Propo:VitC	0	-0.90	NA	-0.90	NA	NA	NA	NA

Supplementary File S5: GRADE Approach analysis and explanations

Criteria used to assess certainty of evidence was based on the Cochrane Training - https://training.cochrane.org/resource/grade-approach-rate-certainty-evidence-network-meta-analysis-and-summary-findings-tables and apropos literature (listed below).

Criteria	Rated down direct estimate if:	Analysis
Risk of bias	When at least one study on the direct comparison presented high risk or some concerns due to problems in the randomization process and lack of blinding.	We rated down the certainty in one level due to some concerns and two levels due to high risk of bias in the randomization process. On the other hand, since glycate hemoglobin is assessed through an automatic laboratory process, no problems with blinding were considered and we did not rate down the certainty.
Inconsistency	• If effect estimates were similar across studies (Guyatt et al. 2011b);<br>• Overlap of 95%Cl (Guyatt et al. 2011b);<br>• I^2 for direct comparisons if was either moderate (30-60%), substantial (50-90%) or considerable (75%-100%) (Schunemann et al., 2021).<br>• If only one study contributed to the direct effect estimate, the certainty was not rated down (Guyatt et al. 2011b).	We did not rate down the certainty for single studies forming the effect estimate. One pair wise meta-analysis included two studies (Omega 3 +NSPT vs NSPT alone -Rampally et al., 2019 and Zare-Javid et al., 2017). As the I^2 was low (0%) chi2 p-value >0.05, effect estimates were similar and there was overlap of 95%CI, we did not rate down the certainty.
Indirectness	Rate down if more than 30% of weight of pooled estimate came from studies which the populations had different characteristics (age, healthy condition) compared to the population of the PICO question (Brignardello-Petersen et al. 2018a).	We assessed indirectness for each comparison. For almost all comparisons, patients had similar characteristics regarding age (late adulthood), T2D and the presence of periodontitis. All patients met the periodontitis case-definition, according to the New Periodontal Diseases Classification (Tonetti et al., 2018). Thus, there was no problems regarding periodontitis condition. The initial glycated hemoglobin (HbA1c) was similar in all populations at the beginning of the study (ranging from 7.3 to 9.9%). However, one study (Zare-Javid et al., 2017) included uncontrolled T2D patients according to the reported glycated hemoglobin (patients had glycated hemoglobin below 6.5% - borderline to T2D diagnosis). Therefore, the comparisons including this study were rated down in one level for indirectness (Omega 3 vs NSPT, Cranberry vs NSPT, Omega 3 + Cranberry vs NSPT, Omega 3 vs Cranberry, Omega 3 vs Omega 3 + Cranberry and Cranberry vs Omega 3 + Cranberry).

Publication bias	If industry funding was associated with a positive outcome favoring the sponsored treatment (Martins et al. 2019).	Lycopene vs NSPT (Reddy et al., 2015) and Omega 3 vs NSPT, Cranberry vs NSPT, Omega 3 + Cranberry vs NSPT, Omega 3 vs Cranberry, Omega 3 vs Omega 3 + Cranberry and Cranberry vs Omega 3 + Cranberry (Zare-Javid et al.; 2017) were industry funded. Yet, no treatment showed superiority when compared to the control, thus we did not rate down due to publication bias.
**Criteria**	**Rated down indirect estimate if:**	**Analysis**
Intransitivity	If there were differences in study characteristics that may modify treatment effect in the direct comparisons that form the basis for the indirect estimate; and if those differences were regarding population, intervention, comparison, or outcome (Puhan et al. 2014).	For intransitivity, we considered the most dominant first order loop, or, in its absence, the closest direct comparison contributing to the effect estimate. Whenever the comparisons contributing to indirect evidence showed important modifications on population or glycated hemoglobin, one point was downgraded due to intransitivity.
**Criteria**	**Rated down NMA estimate if:**	**Analysis**
Incoherence	If node splitting test showed p-value <0.05, and direct, indirect and NMA evidence agreed with each other. (Brignardello-Petersen et al. 2018a).	Node split test was not feasible, due to the poorly connected network. Thus, global inconsistency statistic result was considered to assess incoherence for comparisons including direct and indirect evidence. We rated down the certainty if:<br>- The p-value was >0.05;<br>- Absence of coherence between direct, indirect effect and NMA effect estimates.<br>We rated down in one level if the direct and indirect evidence favored opposite treatments, but the direct effect estimate was in accordance with the NMA effect estimate; and in two levels if the direct effect estimate was not coherent with both the indirect and NMA effect estimates.
Imprecision	A partially contextualized approach was used, considering the large effect threshold according to Cohen's classification (>0.8 SD) (Guyatt et al. 2011a; Schünemann et al. 2021; Brignardello-Petersen et al. 2021).	We rated down in one level if the 95% CI of the NMA effect estimate crossed the large effect threshold; two levels if the NMA 95% CI also crossed the null effect line.<br>In the cases when the threshold was not crossed, the effect size was evaluated. OIS calculations were performed to large unplausible effects. Whenever OIS was not achieved, one point was downgraded (Brignardello-Petersen et al. 2021).

Supplement References

Brignardello-Petersen R, Bonner A, Alexander PE, Siemieniuk RA, Furukawa TA, Rochwerg B, Hazlewood GS, Alhazzani W, Mustafa RA, Murad MH, Puhan MA, Schünemann HJ, Guyatt GH; GRADE Working Group. Advances in the GRADE approach to rate the certainty in estimates from a network meta-analysis. J Clin Epidemiol. 2018 Jan;93:36-44. doi: 10.1016/j.jclinepi.2017.10.005. (a)

Brignardello-Petersen R, Murad MH, Walter SD, McLeod S, Carrasco-Labra A, Rochwerg B, Schünemann HJ, Tomlinson G, Guyatt GH; GRADE Working Group. GRADE approach to rate the certainty from a network meta-analysis: avoiding spurious judgments of imprecision in sparse networks. J Clin Epidemiol. 2019 Jan;105:60-67. Doi: 10.1016/j.jclinepi.2018.08.022.

Brignardello-Petersen R, Mustafa RA, Siemieniuk RAC, Murad MH, Agoritsas T, Izcovich A, Schünemann HJ, Guyatt GH; GRADE Working Group. GRADE approach to rate the certainty from a network meta-analysis: addressing incoherence. J Clin Epidemiol. 2019 Apr;108:77-85. Doi: 10.1016/j.jclinepi.2018.11.025. (b)

Brignardello-Petersen R, Guyatt GH, Mustafa RA, Chu DK, Hultcrantz M, Schünemann HJ, Tomlinson G. GRADE guidelines 33: Addressing imprecision in a network meta-analysis. J Clin Epidemiol. 2021 Nov;139:49-56. doi: 10.1016/j.jclinepi.2021.07.011. Epub 2021 Jul 19.

Guyatt GH, Oxman AD, Kunz R, Brozek J, Alonso-Coello P, Rind D, Devereaux PJ, Montori VM, Freyschussi B, Vist G et al. 2011a. Grade guidelines 6. Rating the quality of evidence -imprecision. J Clin Epidemiol. 64:1283-1293. (b)

Guyatt GH, Oxman AD, Kunz R, Woodcock J, Brozek J, Helfand M, Alonso-Coello P, Glasziou P, Jaeschke R, Akl EA et al. 2011b. Grade guidelines: 7. Rating the quality of evidence--inconsistency. J Clin Epidemiol. 64(12):1294-1302. (a)

Martins CC, Riva JJ, Firmino RT, Colunga-Lozano LE, Granville-Garcia AF, Zhang Y, Schünemann HJ. 2019. Conflict of interest is not associated with positive conclusions in toothpaste trials: A systematic survey. J Clin Epidemiol. 108:140-146.

Puhan MA, Schünemann HJ, Murad MH, Li T, Brignardello-Petersen R, Singh JA, Kessels AG, Guyatt GH; GRADE Working Group. A GRADE Working Group approach for rating the quality of treatment effect estimates from network meta-analysis. BMJ. 2014 Sep 24;349:g5630. doi: 10.1136/bmj.g5630.

Schünemann HJ, Vist GE, Higgins JPT, Santesso N, Deeks JJ, Glasziou P, Akl EA, Guyatt GH. Chapter 15: Interpreting results and drawing conclusions. In: Higgins JPT, Thomas J, Chandler J, Cumpston M, Li T, Page MJ, Welch VA (editors). Cochrane Handbook for Systematic Reviews of Interventions version 6.2 (updated February 2021). Cochrane, 2021. Available from www.training.cochrane.org/handbook.

Tonetti MS, Greenwell H, Kornman KS. Staging and grading of periodontitis: Framework and proposal of a new classification and case definition. J Periodontol. 2018;89(Suppl 1):S159-S172. doi:10.1002/JPER.18-0006

Table S1: Assessment of GRADE for direct and indirect comparisons evidence certainty

Comparison	Number of Studies	Risk of Bias	Inconsistency	Indirectness	Publication Bias	Direct comparison Certainty	Indirect evidence comparison from the closest loop	Indirect evidence comparison from the closest loop	Lowest between both	Intransitivity	Incoherence for NMA	Imprecision for NMA	Final Certainty
**DIRECT COMPARISONS**													
ALA vs NSPT	1	-1 (some conc)	0	0	0	Moderate	-	-	-	-	-	-1	Low
Ginger vs NSPT	1	0	0	0	0	High	-	-	-	-	-	-2	Low
GrapeS vs NSPT	1	-2 (high)	0	0	0	Low	-	-	-	-	-	-2	Very Low
Lycop vs NSPT	1	-1 (some conc)	0	0	0	Moderate	-	-	-	-	-	-2	Very Low
Melato vs NSPT	1	-1 (some conc)	0	0	0	Moderate	-	-	-	-	-	-1	Low
Omega3 vs NSPT	2	-1 (some conc)	0	-1	0	Low	-	-	-	-	-	-2	Very Low
Propol vs NSPT	1	0	0	0	0	High	-	-	-	-	-	-1	Moderate
VitC vs NSPT	1	-1 (some conc)	0	0	0	Moderate	-	-	-	-	-	-2	Very Low
Cranb vs Cranb + Om3	1	-1 (some conc)	0	-1	0	Low	-	-	-	-	-	-2	Very Low
**INDIRECT ONLY COMPARISONS**													
ALA vs Cranb	-	-	-	-	-	-	NSPT vs ALA (Mod)	NSPT vs Cranb (Low)	Low	-1	-	-1	Very Low
ALA vs CranbOm 3	-	-	-	-	-	-	NSPT vs ALA (Mod)	NSPT vs CranbOm3 (Low)	Low	-1	-	-1	Very Low

Comparison	Number of Studies	Risk of Bias	Inconsistency	Indirectness	Publication Bias	Direct comparison Certainty	Indirect evidence comparison from the closest loop	Indirect evidence comparison from the closest loop	Lowest between both	Intransitivity	Incoherence for NMA	Imprecision for NMA	Final Certainty
ALA vs Ginger	-	-	-	-	-	-	NSPT vs ALA (Mod)	NSPT vs Ginger (High)	Mod	0	-	-1	Low
ALA vs GrapeS	-	-	-	-	-	-	NSPT vs ALA (Mod)	NSPT vs Grape (Low)	Low	0	-	-1	Very Low
ALA vs Lycop	-	-	-	-	-	-	NSPT vs ALA (Mod)	NSPT vs Lycop (Mod)	Mod	0	-	-1	Low
ALA vs Melato	-	-	-	-	-	-	NSPT vs ALA (Mod)	NSPT vs Melato (Mod)	Mod	0	-	-2	Very Low
ALA vs Omega3	-	-	-	-	-	-	NSPT vs ALA (Mod)	NSPT vs Omega 3 (Low)	Low	-1	-	-1	Very Low
ALA vs Propo	-	-	-	-	-	-	NSPT vs ALA (Mod)	NSPT vs Propol (High)	Mod	0	-	-1	Low
ALA vs VitC	-	-	-	-	-	-	NSPT vs ALA (Mod)	NSPT vs Vit C (Mod)	Mod	0	-	-1	Low
Cranb vs Ginger	-	-	-	-	-	-	NSPT vs Cranb (Low)	NSPT vs Ginger (High)	Low	-1	-	-2	Very Low
Cranb vs GrapeS	-	-	-	-	-	-	NSPT vs Cranb (Low)	NSPT vs Grape (Low)	Low	-1	-	-2	Very Low
Cranb vs Lycop	-	-	-	-	-	-	NSPT vs Cranb (Low)	NSPT vs Lycop (Mod)	Low	-1	-	-2	Very Low
Cranb vs Melato	-	-	-	-	-	-	NSPT vs Cranb (Low)	NSPT vs Melato (Mod)	Low	-1	-	-2	Very Low
Cranb vs Propo	-	-	-	-	-	-	NSPT vs Cranb (Low)	NSPT vs Propol (High)	Low	-1	-	-2	Very Low
Cranb vs VitC	-	-	-	-	-	-	NSPT vs Cranb (Low)	NSPT vs Vit C (Mod)	Low	-1	-	-2	Very Low

Comparison	Number of Studies	Risk of Bias	Inconsistency	Indirectness	Publication Bias	Direct comparison Certainty	Indirect evidence comparison from the closest loop	Indirect evidence comparison from the closest loop	Lowest between both	Intransitivity	Incoherence for NMA	Imprecision for NMA	Final Certainty
CranbOm 3 vs Ginger	-	-	-	-	-	-	NSPT vs Cranb+Om3 (Low)	NSPT vs Ginger (High)	Low	-1	-	-2	Very Low
CranbOm 3 vs GrapeS	-	-	-	-	-	-	NSPT vs Cranb+Om3 (Low)	NSPT vs Grape (Low)	Low	-1	-	-2	Very Low
CranbOm 3 vs Lycop	-	-	-	-	-	-	NSPT vs Cranb+Om3 (Low)	NSPT vs Lycop (Mod)	Low	-1	-	-2	Very Low
CranbOm 3 vs Melato	-	-	-	-	-	-	NSPT vs Cranb+Om3 (Low)	NSPT vs Melato (Mod)	Low	-1	-	-2	Very Low
CranbOm 3 vs Propo	-	-	-	-	-	-	NSPT vs Cranb+Om3 (Low)	NSPT vs Propol (High)	Low	-1	-	-2	Very Low
CranbOm 3 vs VitC	-	-	-	-	-	-	NSPT vs Cranb+Om3 (Low)	NSPT vs Vit C (Mod)	Low	-1	-	-2	Very Low
Ginger vs GrapeS	-	-	-	-	-	-	NSPT vs Ginger (High)	NSPT vs Grape (Low)	Low	0	-	-2	Very Low
Ginger vs Lycop	-	-	-	-	-	-	NSPT vs Ginger (High)	NSPT vs Lycop (Mod)	Mod	0	-	-2	Very Low
Ginger vs Melato	-	-	-	-	-	-	NSPT vs Ginger (High)	NSPT vs Melato (Mod)	Mod	0	-	-2	Very Low
Ginger vs Omega3	-	-	-	-	-	-	NSPT vs Ginger (High)	NSPT vs Omega 3 (Low)	Low	-1	-	-2	Very Low
Ginger vs Propo	-	-	-	-	-	-	NSPT vs Ginger (High)	NSPT vs Propol (High)	High	0	-	-2	Low
Ginger vs VitC	-	-	-	-	-	-	NSPT vs Ginger (High)	NSPT vs Vit C (Mod)	Mod	0	-	-2	Very Low

Comparison	Number of Studies	Risk of Bias	Inconsistency	Indirectness	Publication Bias	Direct comparison Certainty	Indirect evidence comparison from the closest loop	Indirect evidence comparison from the closest loop	Lowest between both	Intransitivity	Incoherence for NMA	Imprecision for NMA	Final Certainty
GrapeS vs Lycop	-	-	-	-	-	-	NSPT vs Grape (Low)	NSPT vs Lycop (Mod)	Mod	0	-	-2	Very Low
GrapeS vs Melato	-	-	-	-	-	-	NSPT vs Grape (Low)	NSPT vs Melato (Mod)	Low	0	-	-1	Very Low
GrapeS vs Omega3	-	-	-	-	-	-	NSPT vs Grape (Low)	NSPT vs Omega 3 (Low)	Low	-1	-	-2	Very Low
GrapeS vs Propo	-	-	-	-	-	-	NSPT vs Grape (Low)	NSPT vs Propol (High)	Mod	0	-	-2	Very Low
GrapeS vs VitC	-	-	-	-	-	-	NSPT vs Grape (Low)	NSPT vs Vit C (Mod)	Mod	0	-	-2	Very Low
Lycop vs Melato	-	-	-	-	-	-	NSPT vs Lycop (Mod)	NSPT vs Melato (Mod)	Mod	0	-	-1	Low
Lycop vs Omega3	-	-	-	-	-	-	NSPT vs Lycop (Mod)	NSPT vs Omega 3 (Low)	Low	-1	-	-2	Very Low
Lycop vs Propo	0	-	-	-	-	-	NSPT vs Lycop (Mod)	NSPT vs Propol (High)	Mod	0	-	-2	Very Low
Lycop vs VitC	0	-	-	-	-	-	NSPT vs Lycop (Mod)	NSPT vs Vit C (Mod)	Mod	0	-	-2	Very Low
Melato vs Omega3	0	-	-	-	-	-	NSPT vs Melato (Mod)	NSPT vs Omega 3 (Low)	Low	-1	-	-1	Very Low
Melato vs Propo	0	-	-	-	-	-	NSPT vs Melato (Mod)	NSPT vs Propol (High)	Mod	0	-	-2	Very Low
Melato vs VitC	0	-	-	-	-	-	NSPT vs Melato (Mod)	NSPT vs Vit C (Mod)	Mod	0	-	-1	Low
Omega3 vs Propo	0	-	-	-	-	-	NSPT vs Omega 3 (Low)	NSPT vs Propol (High)	Low	-1	-	-2	Very Low
Omega3 vs VitC	0	-	-	-	-	-	NSPT vs Omega 3 (Low)	NSPT vs Vit C (Mod)	Low	-1	-	-2	Very Low

Comparison	Number of Studies	Risk of Bias	Inconsistency	Indirectness	Publication Bias	Direct comparison Certainty	Indirect evidence comparison from the closest loop	Indirect evidence comparison from the closest loop	Lowest between both	Intransitivity	Incoherence for NMA	Imprecision for NMA	Final Certainty
Propo vs VitC	0	-	-	-	-	-	NSPT vs Propol (High)	NSPT vs Vit C (Mod)	Mod	0	-	-2	Very Low
**DIRECT AND INDIRECT COMPARISONS**													
Cranb vs NSPT	1	-1 (some conc)	0	-1	0	Low	Cranb vs Omega3 (Low)	Omega3 vs NSPT (Low)	Low	-1	0	-2	Very Low
Cranb + Om3 vs NSPT	1	-1 (some conc)	0	-1	0	Low	Cranb + Om3 vs Omega3(Low)	Omega3 vs NSPT (Low)	Low	-1	-1	-2	Very Low
Cranb vs Omega3	1	-1 (some conc)	0	-1	0	Low	Cranb vs NSPT(Low)	Omega3 vs NSPT (Low)	Low	-1	0	-2	Very Low
Cranb + Om3 vs Omega3	1	-1 (some conc)	0	-1	0	Low	Cranb + Om3 vs NSPT(Low)	Omega3 vs NSPT (Low)	Low	-1	0	-2	Very Low

Supplement References

Brignardello-Petersen R, Bonner A, Alexander PE, Siemieniuk RA, Furukawa TA, Rochwerg B, Hazlewood GS, Alhazzani W, Mustafa RA, Murad MH, Puhan MA, Schünemann HJ, Guyatt GH; GRADE Working Group. Advances in the GRADE approach to rate the certainty in estimates from a network meta-analysis. J Clin Epidemiol. 2018 Jan;93:36-44. doi: 10.1016/j.jclinepi.2017.10.005. (a)

Brignardello-Petersen R, Murad MH, Walter SD, McLeod S, Carrasco-Labra A, Rochwerg B, Schünemann HJ, Tomlinson G, Guyatt GH; GRADE Working Group. GRADE approach to rate the certainty from a network meta-analysis: avoiding spurious judgments of imprecision in sparse networks. J Clin Epidemiol. 2019 Jan;105:60-67. Doi: 10.1016/j.jclinepi.2018.08.022.

Brignardello-Petersen R, Mustafa RA, Siemieniuk RAC, Murad MH, Agoritsas T, Izcovich A, Schünemann HJ, Guyatt GH; GRADE Working Group. GRADE approach to rate the certainty from a network meta-analysis: addressing incoherence. J Clin Epidemiol. 2019 Apr;108:77-85. Doi: 10.1016/j.jclinepi.2018.11.025. (b)

Brignardello-Petersen R, Guyatt GH, Mustafa RA, Chu DK, Hultcrantz M, Schünemann HJ, Tomlinson G. GRADE guidelines 33: Addressing imprecision in a network meta-analysis. J Clin Epidemiol. 2021 Nov;139:49-56. doi: 10.1016/j.jclinepi.2021.07.011. Epub 2021 Jul 19.

Guyatt GH, Oxman AD, Kunz R, Brozek J, Alonso-Coello P, Rind D, Devereaux PJ, Montori VM, Freyschussi B, Vist G et al. 2011a. Grade guidelines 6. Rating the quality of evidence -imprecision. J Clin Epidemiol. 64:1283-1293. (b)

Guyatt GH, Oxman AD, Kunz R, Woodcock J, Brozek J, Helfand M, Alonso-Coello P, Glasziou P, Jaeschke R, Akl EA et al. 2011b. Grade guidelines: 7. Rating the quality of evidence--inconsistency. J Clin Epidemiol. 64(12):1294-1302. (a)

Martins CC, Riva JJ, Firmino RT, Colunga-Lozano LE, Granville-Garcia AF, Zhang Y, Schünemann HJ. 2019. Conflict of interest is not associated with positive conclusions in toothpaste trials: A systematic survey. J Clin Epidemiol. 108:140-146.

Puhan MA, Schünemann HJ, Murad MH, Li T, Brignardello-Petersen R, Singh JA, Kessels AG, Guyatt GH; GRADE Working Group. A GRADE Working Group approach for rating the quality of treatment effect estimates from network meta-analysis. BMJ. 2014 Sep 24;349:g5630. doi: 10.1136/bmj.g5630.

Schünemann HJ, Vist GE, Higgins JPT, Santesso N, Deeks JJ, Glasziou P, Akl EA, Guyatt GH. Chapter 15: Interpreting results and drawing conclusions. In: Higgins JPT, Thomas J, Chandler J, Cumpston M, Li T, Page MJ, Welch VA (editors). Cochrane Handbook for Systematic Reviews of Interventions version 6.2 (updated February 2021). Cochrane, 2021. Available from www.training.cochrane.org/handbook.

Tonetti MS, Greenwell H, Kornman KS. Staging and grading of periodontitis: Framework and proposal of a new classification and case definition. J Periodontol. 2018;89(Suppl 1):S159-S172. doi:10.1002/JPER.18-0006
